# Nanocurvature‐Activated Dipolar Polarization in M–N_4_ Single‐Atom Sites for High‐Performance Electromagnetic Wave Absorption

**DOI:** 10.1002/advs.75115

**Published:** 2026-03-31

**Authors:** Daohu Sheng, Siyao Cheng, Mengmeng Zhang, Jinglei Zhang, Xufei Zhu, Weijin Li, Bo Zhang, Aming Xie

**Affiliations:** ^1^ School of Safety Science and Engineering Nanjing University of Science and Technology Nanjing P. R. China; ^2^ School of Chemistry and Chemical Engineering Nanjing University of Science and Technology Nanjing P. R. China; ^3^ State Key Laboratory of Structural Chemistry Fujian Institute of Research on the Structure of Matter Chinese Academy of Sciences Fuzhou P. R. China; ^4^ MIIT Key Laboratory of Advanced Display Materials and Devices & Materials Physical and Chemical Research and Practice Center College of Materials Science and Engineering Nanjing University of Science and Technology Nanjing P. R. China; ^5^ CAS Key Laboratory of Science and Technology on Applied Catalysis Dalian Institute of Chemical Physics Chinese Academy of Sciences Dalian P. R. China

**Keywords:** carbon based absorbers, dipole polarization, electromagnetic wave absorption, metal single atoms, nanocurvature engineering

## Abstract

The dielectric response of carbon‐based single‐atom (SA) absorbers is intrinsically constrained by the highly symmetric charge distribution of planar M‐N_4_ coordination motifs, which suppresses dipole polarization and limits electromagnetic wave (EMW) attenuation. Here, a nanocurvature‐driven symmetry‐breaking strategy is proposed to activate latent dielectric polarization at SA sites through geometric regulation. By combining click chemistry with template‐assisted synthesis, metal SAs are anchored onto hollow nitrogen‐doped carbon spheres with precisely tunable diameters, enabling systematic modulation of local nanocurvature. Theoretical calculations and experimental analyses reveal that curvature‐induced surface charge accumulation disrupts the electronic symmetry at Ni‐N_4_ centers, markedly enhancing local charge density, dipole moments, and polarizability. Consequently, the optimized Ni/HNC‐200 absorber achieves an ultralow minimum reflection loss of −74.1 dB, representing a staggering 390% enhancement over HNC‐200 (−15.1 dB), and exhibits a reduced radar cross section of −70.49 dB m^2^. A flexible electronic patch further demonstrates over fivefold suppression of electric‐field radiation from mobile phone chips. The universality of this mechanism is validated in Co‐ and Cu‐based systems, establishing nanocurvature as a geometry‐enabled design paradigm for high‐performance SAs EMW absorbers.

## Graphical abstract

Increasing nanocurvature effectively enriches the local electron density of the M–N_4_ motif, which facilitates electron transfer from the metal center to nitrogen atoms. This electronic behavior intensifies the localization and asymmetric distribution of charge, thereby significantly amplifying the dipole moment and polarizability. Consequently, the intrinsic polarization loss characteristics of M–N_4_ dipoles are optimized.

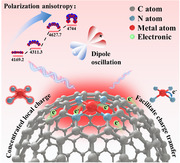

## 1 Introduction

Carbon‐anchored metal single atoms (M‐SAs) have emerged as a frontier platform for electromagnetic wave (EMW) absorption owing to their maximal atomic utilization and highly tunable electronic structures that outperform conventional metal clusters or bulk counterparts [1, 2, 3]. When incorporated into conductive carbon networks, isolated metal centers function as efficient polarization units, enabling strong dielectric relaxation that is essential for next‐generation intelligent electromagnetic devices [4, 5]. Despite these advantages, the performance of carbon‐based SA absorbers remains fundamentally constrained by a prevailing structural motif, namely planar M‐N_4_ coordination, which predominantly accommodates small radius fourth period transition metals [6, 7]. In such configurations, metal atoms are embedded within the graphene plane, producing highly symmetric charge distributions [8] that intrinsically suppress dipole polarization and consequently limit dielectric loss and EMW attenuation efficiency. This intrinsic conflict between coordination stability and polarization activity represents a persistent bottleneck in the rational design of SA absorbers.

To address this limitation, substantial efforts have been devoted to perturbing the local coordination environment of M‐SAs. Approaches including defect engineering [9, 10], heteroatom substitution (e.g., N, O, S, P) [11, 12], axial coordination using halogen ligands (e.g., F, Cl) [13, 14], and nonplanar coordination geometries [15] have been employed to disrupt the planar M‐N_4_ symmetry and enhance dielectric losses. Although these strategies have achieved partial improvements, they are largely empirical and often suffer from stochastic atomic distributions and limited geometric uniformity, leaving a considerable fraction of SA sites electronically unmodulated. Moreover, the coexistence of multiple loss mechanisms, including interfacial polarization, conduction loss, and magnetic loss, obscures the intrinsic contribution of atomic‐scale dipole polarization [16, 17]. As a result, a rational design strategy that enables precise geometric regulation of SA coordination sites while establishing a clear structure polarization performance relationship remains elusive.

**FIGURE 1:**
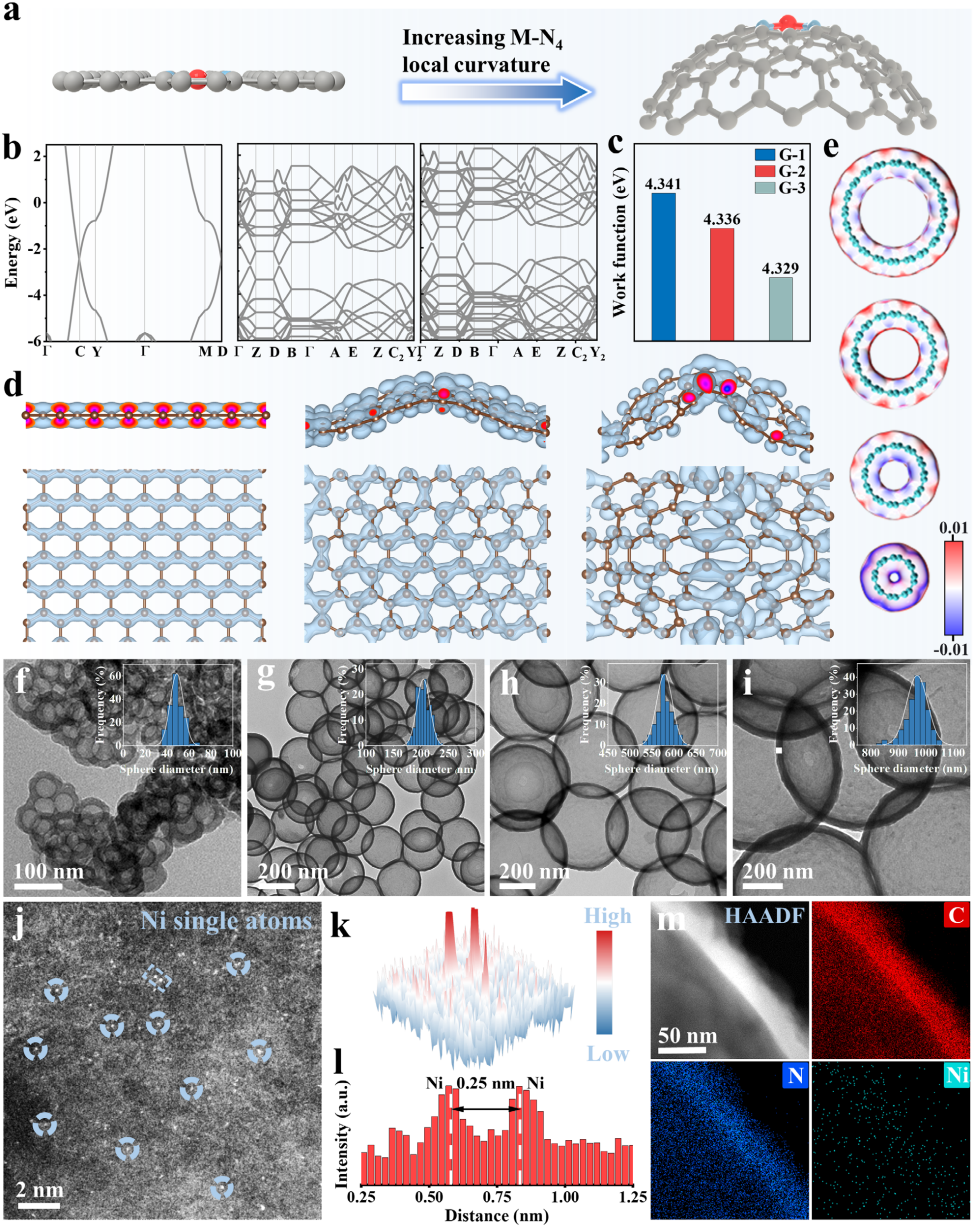
Density functional theory calculations, absorber design, and materials characterization. (a) Schematic diagram of the research on the dielectric properties of metal‐N_4_ within a carbon lattice with Nanocurvature. (b) Band structures (From left to right, they are G‐1, G‐2, G‐3; the curvature of graphene progressively increases), (c) work function, and (d) charge density of frontier molecular orbitals of curved graphene with various curvatures. (e) Electrostatic potential of the carboatomic ring with various curvatures. TEM images of (f) Ni/HNC‐50, (g) Ni/HNC‐200, (h) Ni/HNC‐580, and (i) Ni/HNC‐970. The inset of each image shows the size distribution of the carbon nanospheres. More TEM images are shown in the Supporting Information. (j) AC‐HAADF‐STEM image of Ni/HNC‐200. (k) 3D atomic overlapping Gaussian‐fitted map and (l) intensity profile of the atomic sites within the selected region of j. (m) HAADF‐STEM and corresponding EDS mapping images of Ni/HNC‐200.

Nanocurvature engineering provides a fundamentally distinct and underexplored pathway by introducing geometric asymmetry as an independent physical design parameter (Figure 1a). Curvature‐induced structural deformation can generate localized strain fields and redistribute surface charges, thereby perturbing the electronic symmetry of atomically dispersed metal centers (Table S1) [18, 19, 20, 21]. For planar M‐N_4_ coordination motifs in particular, nanocurvature is expected to induce out‐of‐plane charge polarization and enhance dipole activity in a geometry‐dependent manner. Although recent studies have begun to incorporate curved architectures into EMW absorbers, systematic understanding remains limited [22, 23]. The fabrication of structures with uniform curvature remains challenging, resulting in spatially heterogeneous electromagnetic responses. Simultaneously, the complexity of multicomponent systems introduces various synergistic and competitive loss mechanisms. This complexity renders it difficult to effectively decouple and quantify the specific contribution of nanocurvature to polarization effects, thus precluding the use of nanocurvature as an independent physical descriptor for elucidating the mechanism of polarization enhancement. Addressing these issues requires a methodology that ensures geometric uniformity and isolates the specific polarization loss mechanism.

Here, we integrate a click chemistry strategy with a template‐assisted synthesis approach to anchor M‐SAs onto hollow nitrogen‐doped carbon spheres with precisely tunable diameters. This dual strategy ensures uniform and monodisperse dispersion of metal atoms while enabling systematic control of nanocurvature through regulation of shell size. By minimizing magnetic, interfacial, and conduction losses, the EMW attenuation mechanism is predominantly attributed to curvature‐modulated Ni‐N_4_ dipole polarization. Experimental results demonstrate that increasing nanocurvature significantly enhances dielectric polarization loss at Ni–N_4_ sites. Density functional theory calculations further reveal that nanocurvature‐driven electronic redistribution increases local charge density, electron localization, and spatial charge inhomogeneity, collectively amplifying dipole moments and polarizability. As a result, the optimized Ni/HNC‐200 absorber achieves an ultralow minimum reflection loss (RL_min_) −74.1 dB, representing a substantial enhancement over its planar analogue. Nanocurvature‐dependent polarization enhancement is further validated in Co‐ and Cu‐based SA systems, establishing nanocurvature as a geometry‐enabled design paradigm for regulating atomic‐scale polarization in high‐performance EMW absorbers.

## 2 Results and Discussion

To examine the impact of nanocurvature on the electronic structure of carbon substrates, three graphene models with varying curvatures were constructed (Figure S1). Simultaneously, their band structures and work functions were calculated. Compared to planar graphene, the high‐symmetry K‐point path in curved graphene shifts to Γ‐Z‐D‐B‐Γ‐A‐E‐Z‐C_2_‐Y_2_ (Figure 1b), and the conduction band moves downward near the Fermi level. These changes indicate that nanocurvature significantly influences the electronic structure of graphene. Work function results reveal a significant decrease with increasing nanocurvature (Figure 1c). This reduction weakens electron binding and enhances surface band bending, facilitating the migration of internal charges to the surface. For semiconductor materials, this phenomenon promotes surface dipole formation, thereby enhancing EMW absorption performance.

Further analysis of the electronic states near the Fermi level, including the conduction band minimum (CBM) and valence band maximum (VBM). The results indicate that in planar graphene, these states are dominated by delocalized π‐bonding and π^*^ antibonding orbitals. In curved graphene, although the overall character of the CBM and VBM remains similar, the introduction of curvature disrupts the coupling between the extended π‐network states. This decoupling leads to increased localization of electrons in the frontier molecular orbitals, as visualized in Figure 1d and Figure S2. Gaussian mathematical derivations indicate that charges preferentially accumulate on positively curved surfaces (Figure S3) [23]. Further electrostatic potential calculations for carbon rings of different diameters confirm that higher curvature leads to greater electron enrichment on the surface (Figure 1e), consistent with the above analysis. Elevated surface electron density fosters the accumulation of electric dipoles, positively influencing EMW absorption [24, 25].

Encouraged by these findings, the modulation of nanocurvature on Ni–N_4_ dipoles was investigated through experimental research. Hollow polypyrrole (HPPy) nanospheres with controlled diameters were synthesized via a template‐assisted method [26]. Subsequently, a click‐confinement strategy was employed to covalently graft carboxyl‐functionalized nickel porphyrin (NiPor) onto the secondary amine groups of the HPPy surface through a carbonyl‐to‐amide conversion reaction (Figure S4). The successful covalent immobilization of NiPor on the HPPy was confirmed by Fourier‐transform infrared (FTIR) spectroscopy, where the emergence of the amide I band (νC═O) at 1649 cm^−1^ explicitly verifies the formation of amide linkages (Figure S5) [27]. Pyrolysis under an argon atmosphere was then conducted to carbonize the HPPy nanospheres into hollow nitrogen‐doped carbon (HNC) nanospheres, simultaneously generating atomically dispersed M–N_4_ coordination sites. The synthesized absorbers are designated as “M/HNC‐X,” where M denotes the transition metal species (Ni, Cu, Co) and X represents the nominal diameter (in nm) of the hollow carbon spheres.

The synthesized Ni/HNC‐X absorbers with varying diameters were characterized using scanning electron microscopy (SEM) and transmission electron microscopy (TEM). SEM images revealed uniform, well‐defined spherical morphologies for the Ni absorbers (Figures S6 and S7). Statistical analysis of approximately 150 particles from SEM and TEM images yielded average diameters of 50, 200, 580, and 970 nm. Representative TEM images of the corresponding absorbers, which are denoted as Ni/HNC‐50, Ni/HNC‐200, Ni/HNC‐580, and Ni/HNC‐970 (Figure 1f–i). Size distribution histograms, included as insets in each TEM image, confirm the narrow dispersity of the spherical architectures. Furthermore, the hollow nature of these EMW absorbers was clearly observable, with no visible Ni nanoparticles or clusters detected, as further supported by Figures S8 and S9.

The X‐ray diffraction (XRD) patterns of all Ni/HNC‐X absorbers display only two broad peaks corresponding to graphitic carbon, with no detectable metallic Ni phases, confirming the absence of crystalline Ni (Figure S10). Aberration‐corrected high‐angle annular dark‐field scanning transmission electron microscopy (AC‐HAADF‐STEM) images further confirm the uniform dispersion of isolated Ni single atoms on the carbon matrix (Figure 1j; Figure S11). A magnified 3D Gaussian‐fitted atomic map of the region in Figure 1j clearly confirms the atomic dispersion of Ni species (Figure 1k). Line profile analysis further reveals an interatomic spacing of ∼0.25 nm (Figure 1l), excluding metallic Ni aggregation. These results validate the efficacy of the click strategy in suppressing metal agglomeration during pyrolysis. Energy‐dispersive X‐ray spectroscopy (EDS) elemental mappings confirm the homogeneous distribution of C, N, and Ni across the synthesized samples (Figure 1m; Figure S12). Inductively coupled plasma optical emission spectroscopy (ICP‐OES) quantifies the Ni loading in each Ni/HNC‐X sample, revealing comparable Ni loadings of approximately 0.1 wt.% (Figure S13 and Table S2).

**FIGURE 2:**
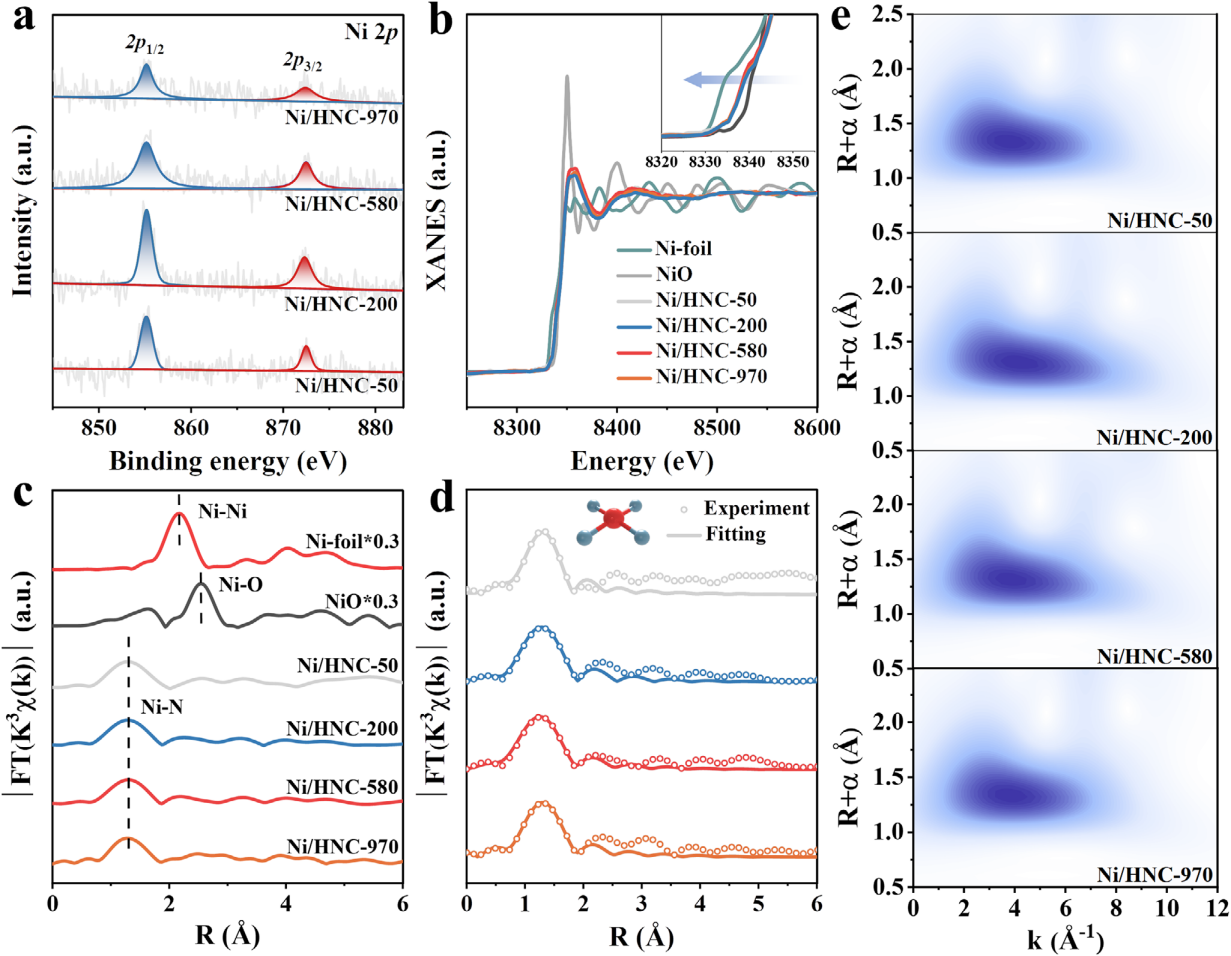
X‐ray spectroscopic characterizations. (a) High‐resolution XPS spectra of Ni 2*p* for Ni/HNC‐X (X = 50, 200, 580, 970). (b) XANES Ni K‐edge spectrum, and localized magnification of XANES Ni K‐edge spectrum. (c) Fourier transform EXAFS spectra, and (d) EXAFS fitting results of Ni/HNC‐X (X = 50, 200, 580, 970), legend color is the same as that in 2c. (e) Wavelet transform (WT) of Ni/HNC‐X (X = 50, 200, 580, 970).

The electronic structure and local coordination environment of the samples were subsequently probed by Raman spectroscopy and X‐ray photoelectron spectroscopy (XPS). Raman spectra revealed two characteristic peaks at 1340 cm^−1^ (*D* band) and 1576 cm^−1^ (*G* band), corresponding to structural disorder and graphitic domains, respectively (Figure S14). All samples exhibited similar *I_D_/I_G_* ratios (∼1.05), indicating comparable graphitization. XPS survey spectra confirmed the presence of C, N, and Ni. High‐resolution N 1s spectra of were deconvoluted into four components: pyridinic N (398.3 eV), Ni─N coordination (399.6 eV), pyrrolic N (400.8 eV), and graphitic N (402.0 eV) (Figure S15). The relative nitrogen species contents remained consistent across all Ni/HNC‐X samples, regardless of particle size (Figure S16 and Table S3). Notably, the Ni 2*p*_3_/_2_ binding energy centered at 855.1 eV falls between metallic Ni^0^ (852.6 eV) and Ni^2^^+^ (855.7 eV) [28], suggesting an intermediate oxidation state (Figure 2a).

Ni K‐edge X‐ray absorption near‐edge structure (XANES) spectra elucidated the atomic‐scale structural of Ni species in Ni/HNC‐X. As shown in Figure 2b, all Ni/HNC‐X samples exhibit nearly identical K‐edge positions, located between those of Ni foil (Ni^0^) and NiO (Ni^2^^+^), indicating an intermediate oxidation state, consistent with XPS results. Extended X‐ray absorption fine structure (EXAFS) spectra reveal a primary peak at 1.30 Å, assigned to the Ni–N first coordination shell (Figure 2c) [29, 30]. Notably, no Ni–Ni scattering peaks (∼2.16 Å) are detected, confirming atomic dispersion of Ni as isolated sites, rather than clusters or multi‐atom ensembles, which is consistent with electron microscopy observations. Least‐squares EXAFS fitting (Figure 2d; Table S4) yields an average Ni–N coordination number of ∼4, consistent with Ni–N_4_ moieties. Wavelet transform (WT)‐EXAFS analysis, which provides simultaneous resolution in k‐spaces and R‐spaces, further confirms these findings. The WT contour plots show a dominant intensity at ∼3.9 Å^−1^, attributed to Ni–N coordination [29], with no discernible signals from Ni–Ni or Ni–O contributions (Figure 2e), further verifying the atomic dispersion of Ni across all samples.

**FIGURE 3:**
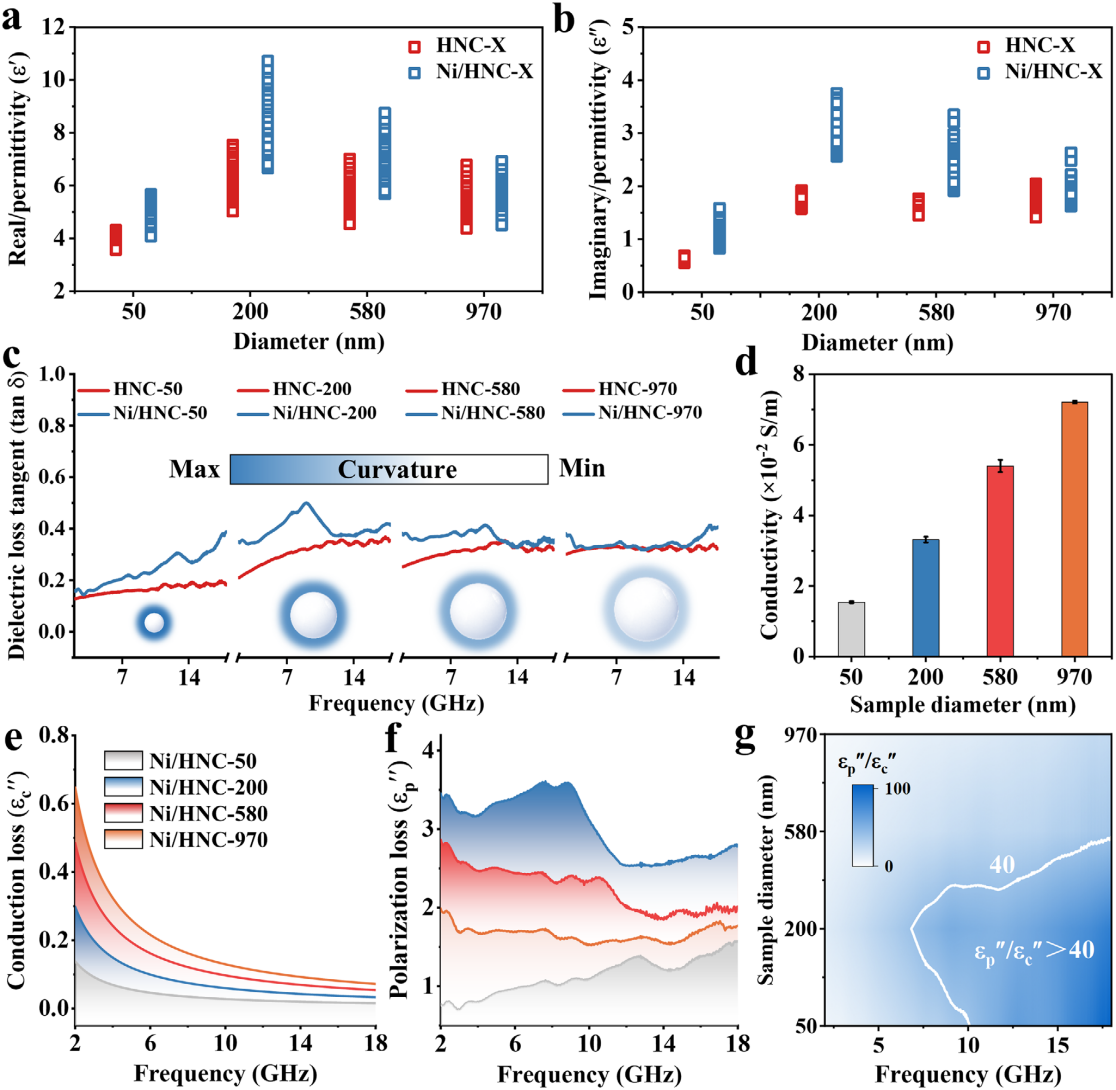
Effect of nanocurvature on dielectric properties. (a) Real and (b) imaginary part of complex permittivity. (c) Dielectric loss tangent, (d) electrical conductivity of samples dispersed in paraffin wax, (e) conduction loss, (f) polarization loss, and (g) polarization loss/conduction loss ratios of Ni/HNC‐X (X = 50, 200, 580, 970).

To assess the influence of nanocurvature‐induced field effects on EMW absorption performance, the complex permittivity (*ε*_r_ = *ε*׳–*jε*״) and permeability (*µ*_r_ = *µ*׳−*jµ*״) of all samples were systematically measured (Table S5). Samples with higher curvature showed stronger electric field responses and greater dielectric loss (Figure 3a,b). Among the Ni/HNC‑50/200/580/970 series, Ni/HNC‐50 exhibited reduced dielectric performance, attributed to discontinuous conductive networks arising from its higher density and lower filler loading at a fixed volume fraction [31]. Given the ultralow metal atomic loading (∼0.1 wt%), the real permeability (µ′) remained near 1 and imaginary permeability (µ″) near 0 in all samples (Figure S17), indicating negligible magnetic loss. Therefore, EMW absorption in Ni/HNC‐X is predominantly mediated by dielectric loss. The dielectric loss tangent (tan δ_ε_ = ε″/ε′), which quantifies dielectric dissipation, increased significantly with curvature (Figure 3c). This trend confirms that the electronic effects induced by nanocurvature effectively enhance dielectric loss capability.

Dielectric loss in EMW absorbers comprises both conduction loss and polarization loss [32]. To clarify nanocurvature‐induced enhancements, Debye theory was applied to decouple conduction loss (*ε_c_* ″) from polarization loss (*ε*_p_″) [33, 34]. The electrical conductivities of Ni/HNC‐50, Ni/HNC‐200, Ni/HNC‐580, and Ni/HNC‐970 dispersed in paraffin were measured via the four‐probe method, yielding values of 0.0154, 0.0332, 0.0540, and 0.0721 S/m, respectively (Figure 3d). According to the Drude model, where conductivity governs free‐carrier‐driven conduction loss [32, 35], these low conductivities result in negligible *ε_c_* ″ contributions (Figure 3e). Polarization losses include dipolar, interfacial, ionic, and electronic polarization [36, 37]. Ionic and electronic relaxation at microwave frequencies (10^3^–10^6^ GHz) were ruled out due to their negligible activity in the 2–18 GHz range [38]. Interface and defect polarization contributions from the carbon matrix were also deemed negligible given the low absorption of the original HNC sample and absence of heterogeneous interfaces (Figure S18). Consequently, the performance improvement is predominantly attributed to Ni–N_4_ dipole polarization, which intensifies with increased curvature (Figure 3f). Remarkably, in Ni/HNC‐50 and Ni/HNC‐200, polarization loss exceeds conduction loss by more than 40‐fold across a wide frequency range (Figure 3g), confirming the substantial enhancement of dipole‐polarization loss at Ni–N_4_ sites via nanocurvature‐induced electronic modulation.

Electron paramagnetic resonance (EPR) spectroscopy was used to probe the Ni–N_4_ dipole polarization under nanocurvature modulation. A single symmetric signal peak (Figure S19) indicates the presence of unpaired electrons [17, 39]. The intensity of this peak is proportional to the concentration of unpaired spins, reflecting the amount of surface‐dispersed electrons that can participate in dipole polarization under an alternating EM field. Furthermore, increased curvature was found to reduce the electrochemical charge‐transfer impedance (Figure S20), lowering energy barriers and facilitating charge migration [40, 41]. This enhanced conductivity promotes Ni‐site dipole polarization under EM excitation. Together, these results demonstrate that nanocurvature increases the density of unpaired electrons and improves charge mobility, thereby enhancing Ni–N_4_ dipole polarization and contributing fundamentally to improved dielectric loss in the absorber.

**FIGURE 4:**
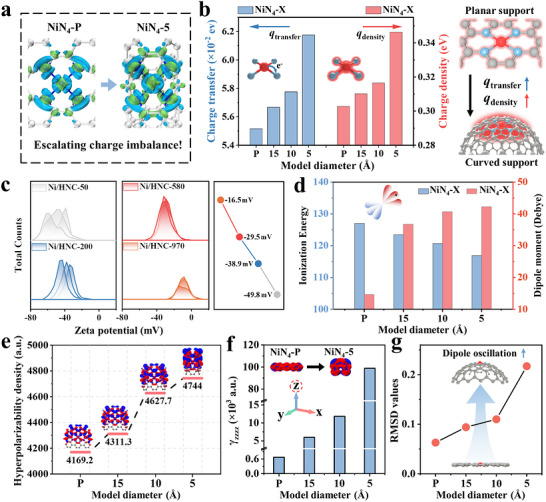
Density functional theory calculations. (a) Differential charge density plots of NiN_4_‐P and NiN_4_‐5. (b) The amount of charge transferred from Ni to N, and Hirshfeld charge calculations of Ni‐N_4_ (The right panel illustrates the electron distribution of the M–N_4_ moiety on planar and curved support). (c) Zeta potential characterizes Ni/HNC‐X (X = 50, 200, 580, 970). (d) Ionization energy and dipole moment of NiN_4_‐P, NiN_4_‐15, NiN_4_‐10, and NiN_4_‐5. (e) Calculated hyperpolarizability of NiN_4_‐P, NiN_4_‐15, NiN_4_‐10, and NiN_4_‐5 (The illustrations depict the local hyperpolarizability contributions of NiN_4_‐P, NiN_4_‐15, NiN_4_‐10, and NiN_4_‐5, with red and blue regions representing positive and negative spatial contributions, respectively), and (f) z‐component of calculated second‐order hyperpolarizability (The illustrations depict the local hyperpolarizability contributions of NiN_4_‐P and NiN_4_‐5). (g) Root mean square displacement (RMSD) plot.

To clarify how nanocurvature enhances Ni–N_4_ dipole polarization at the atomic scale, systematic DFT calculations were carried out on graphene‐based Ni–N_4_ models with increasing curvature (Figure S21). Differential charge density maps reveal that electrons accumulate preferentially at Ni and adjacent N atoms. Moreover, compared to the planar Ni‐N_4_, the nanocurvature‐induced Ni‐N_4_ exhibits more pronounced local electron cloud distortion with charge transfer from Ni to N increasing as curvature increases (Figure 4a,b; Figure S22). Relative to the planar reference, local nanocurvature amplifies charge imbalance and promotes electronic polarization of the Ni–N_4_ centers. Hirshfeld population analysis shows that both Ni and N atoms gain valence electron density as curvature increases (Table S6), revealing active electron enrichment in the Ni–N_4_ motif (∼+ 0.303 to 0.346 eV). When comparing fixed surface regions across models, electron count consistently increases with curvature (Figure 4b), confirming a spatially inhomogeneous charge distribution. Experimentally, we characterized the surface potential properties of all samples using Zeta potential analysis. The Zeta potential spectra revealed that high‐curvature samples exhibit multiple overlapping broad peaks, indicating a more heterogeneous surface charge distribution. This result demonstrates that increased nanocurvature enhances the surface charge imbalance in the samples. Furthermore, zeta potential measurements show a progressive surface potential shift from −28.4 to −56.7 mV as curvature increases (Figure 4c), indicating that electrons are preferentially transferred to the exterior surface [42, 43]. This behavior aligns with literature accounts linking surface curvature and electrokinetic charge distribution in carbon systems. Additionally, calculated ionization energies of Ni atoms decrease systematically with increasing curvature (Figure 4d), indicating that electron‐enriched Ni sites facilitate more efficient electron transfer to neighboring N atoms. Theoretical calculations and experimental studies demonstrate that greater nanocurvature induces stronger charge imbalance around M–N_4_ sites. Together, these findings show that nanocurvature significantly increases local electron density at Ni–N_4_ sites by promoting electron localization and generating strong electrostatic polarization. The resulting spatial charge inhomogeneity enhances the polarizability, enabling more electrons to engage in Ni–N_4_ dipole relaxation under an alternating EM field.

Charge imbalance is known to increase the dipole moment, and according to dielectric theory, polarization loss scales positively with dipole moment [22]. To quantify this effect, the dipole moments of all Ni/HNC‐X models were calculated. As shown in Figure 4d, dipole magnitude increases systematically across Ni/HNC‐50, ‐200, ‐580 to ‐970, in line with increasing curvature. This trend reflects enhanced charge transfer under curved geometries and corresponds to greater distortion in intrinsic charge density and local electric fields, facilitating both carrier separation and polarization field formation. Hyperpolarizability density analysis enables visualization of the spatial contributions from different molecular regions to the overall electrical response, reflecting the nonlinear polarization behavior of the system [44, 45]. Figure 4e displays the hyperpolarizability density distribution of models with different nanocurvature under a static electric field, illustrating the local electron density contributions to the hyperpolarizability (Figure S23). Notably, the hyperpolarizability increases linearly with curvature, with the highly curved NiN_4_‐5 structure reaching 4744.0 a.u., which significantly exceeds the value of the planar NiN_4_‐P structure at 4169.2 a.u. This marked difference indicates enhanced polarization anisotropy in the curved system. Component analysis further reveals that the hyperpolarizability component perpendicular to the structural plane increases exponentially with curvature (Figure 4f; Figure S24 and Table S7). This behavior likely stems from curvature‐induced electron localization, where delocalized electrons transition to localized states, thereby enhancing the polarization response of the local Ni‐N_4_ structure. Collectively, these hyperpolarizability analyses confirm a distinct curvature‐dependent enhancement of polarization at local Ni‐N_4_ sites.

Electric‐field‐driven dynamics further corroborate these findings: the root‐mean‐square displacement (RMSD) of the Ni–N_4_ dipole under applied field increases with curvature (0.05< 0.094< 0.110< 0.217 nm), providing direct evidence of intensified dipole vibration. (Figure 4g; Movies S1–S4). In tandem with these atomistic insights, the Cole–Cole plots reveal progressively larger and more well‐defined semicircular arcs for high‐curvature samples (Figure S25), indicating stronger relaxation polarization consistent with Debye‐based DFT trends. Nonlinear least‐squares fitting to the corrected Debye model affirms that polarization loss extracted from these curves aligns closely with both experimental and theoretical data (Figure S26). Based on experimental and theoretical calculations, nanocurvature‐induced electron enrichment explains the observed enhancement in polarization loss. Higher curvature promotes surface charge accumulation and increases the local electron density at Ni–N_4_ sites. This effect intensifies charge localization and spatial inhomogeneity, in turn amplifying dipole moment and polarizability. As a result, the intrinsic polarization loss characteristics of individual Ni–N_4_ dipoles are optimized, leading to markedly improved dielectric dissipation in high‐curvature samples. This mechanism establishes a quantitative, atomic‐scale framework for designing high‐performance EMW absorption materials with superior dipolar loss.

**FIGURE 5:**
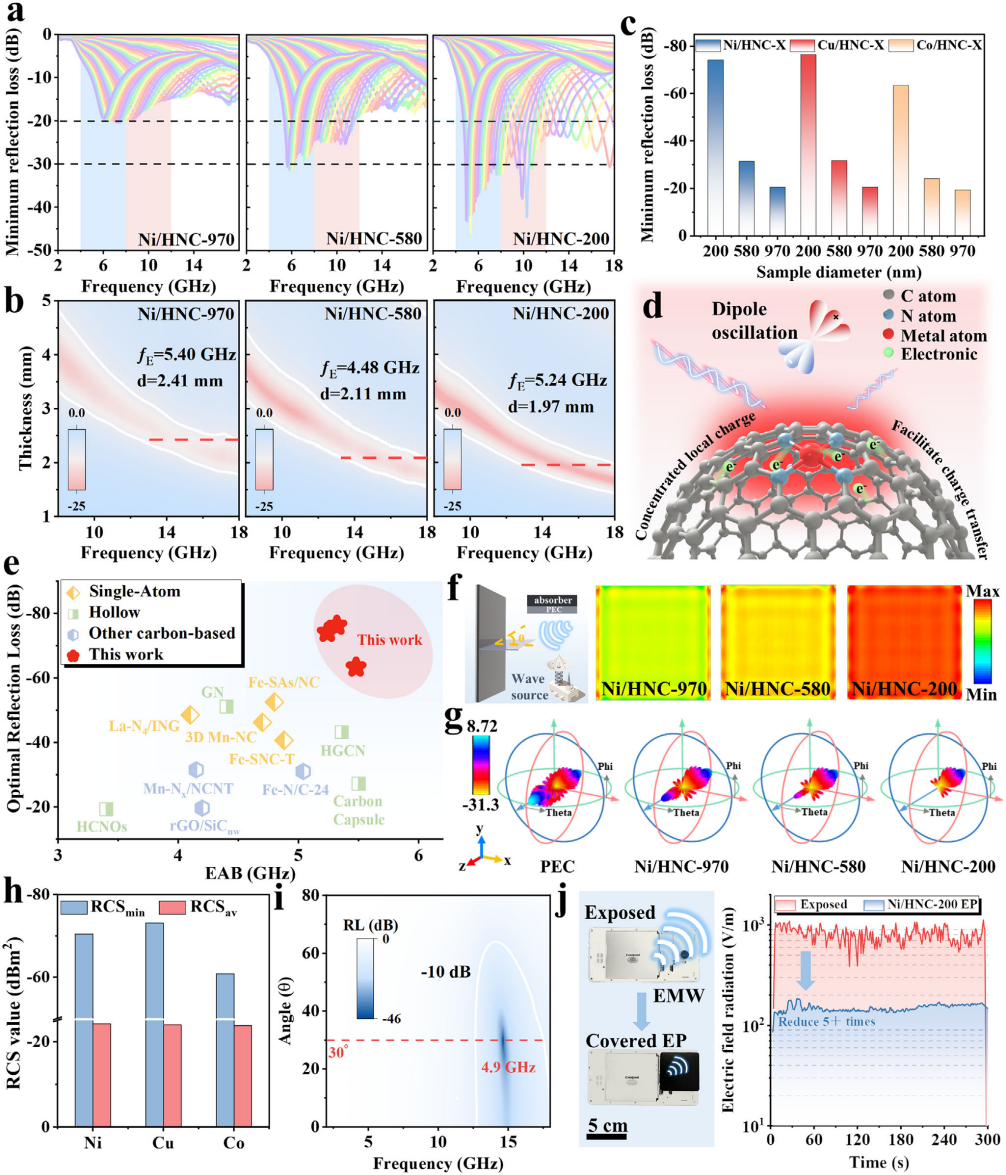
EMW absorption performance and applications. (a) RL values at various thicknesses (matching thickness: 1–5 mm; Increment: 0.1 mm). (b) 2D RL plots of the Ni/HNC‐X (X = 200, 580, 970). (c) Summary of RL_min_ of the samples with different metal single atoms. (d) M‐N_4_ polarization loss enhancement mechanism. (e) Compared with the EMW absorption performance of other carbon materials with special structures. (f) Surface current distributions of Ni/HNC‐X (X = 200, 580, 970). (g) RCS simulation of PEC and Ni/HNC‐X (X = 200, 580, 970). (h) RCS values of M/HNC‐200 (M = Ni, Cu, Co). (i) EMW absorption performance of Ni/HNC‐200 at different angles of incidence of EMW. (j) E‐field radiation values before and after applying Ni/HNC‐200 EP.

The EMW absorption properties of samples with varying curvature were determined using transmission‐line theory. Minimum reflection loss (RL_m__i__n_) is the key performance index in this field (RL ≤ −10 dB corresponds to ≥ 90% incident‐EMW absorption). Obviously, with the increase in nanocurvature, the enhanced Ni‐N_4_ dipole polarization significantly improved the RL of the samples (Figure 5a,b; Figure S27). Significantly, Ni/HNC‐200 exhibits exceptional absorption performance, maintaining a reflection loss (RL) surpassing −20 dB across a wide thickness range of 1–5 mm. Particularly in the C and X bands, RL values predominantly reach −30 dB at most thicknesses, culminating in a minimum of −74.1 dB at 10.12 GHz. This represents a striking 261.5% enhancement compared to the relatively planar reference, Ni/HNC‐970 (−20.5 dB). To validate the generality of nanocurvature‐induced electronic effects on EMW absorption enhancement, we synthesized analogs with Cu and Co metal centers. These exhibited similar performance trends and significantly improved absorption rates (Figures S28–S32). Among them, Cu/HNC‐200 and Co/HNC‐200 achieved RL_min_ values of −76.3 dB at 8.96 GHz and −63.3 dB at 6.52 GHz, respectively (Figure 5c). Since ε_c_″ = σ/(2πfε_0_), conduction loss diminishes sharply with frequency, in materials with moderate conductivity (σ ≈ 10^−3^–10^−1^ S/m), conduction loss is only significant below ∼1–2 GHz [46]. As the observed RL_m__i__n_ enhancement falls entirely within the 2–18 GHz range, the dominant attenuation is attributed to nanocurvature‐induced amplification of M–N_4_ dipole polarization loss, rather than conductive mechanisms (Figure 5d). Consequently, compared to state‐of‐the‐art carbon‐based absorbers with tailored architectures (e.g., single‐atom, hollow structures, and hybrid composites), our curvature‐engineered M/HNC‐X exhibits superior EMW attenuation efficiency, as quantitatively validated in Figure 5e and Table S8.

The effectiveness of nanocurvature‐enhanced M‐N_4_ polarization loss was validated through radar cross‐section (RCS) simulations. We constructed a simulation model of a metal backplane coated with an absorbing layer and performed surface current distribution analysis. The results indicate that, compared to other samples, the Ni/HNC‐200 coated model exhibits higher surface current density and broader distribution (Figure 5f), demonstrating its ability to effectively induce eddy currents and enhance the interaction between current and EMW, thereby improving energy dissipation efficiency. Consequently, the Ni/HNC‐200‐coated model shows weaker EM scattering characteristics (Figure 5g), indicating that Ni/HNC‐200 possesses superior EMW absorption performance and stronger reflection loss in practical applications, consistent with the surface current simulation results. Further, we simulated the RL trend at dynamically tuned frequencies and verified its strong electromagnetic radiation dissipation characteristics through 3D RCS color map visualization. The pure perfect electrical conductor (PEC) model exhibited strong scattering at every frequency point, whereas the EMW absorber‐coated model demonstrated frequency‐dependent low RCS behavior (Figures S33–S39). These low‐RCS frequencies aligned with the reflection loss patterns calculated using transmission line theory (Figure S40). Notably, Ni/HNC‐200, Cu/HNC‐200, and Co/HNC‐200 achieved ultralow RCS values of −70.49, −73.15, and −60.81 at 10.12, 8.96, and 6.52 GHz, respectively (Figure 5h; Table S9). This indicates virtually undetectable scattering signals, demonstrating complete absorption and dissipation of electromagnetic waves.

The metal‐backed absorbers coated with the optimized material achieved radar cross‐sections (RCS) below −20 dBm^2^ across a broad scanning angle range (−90° to 90°) (Figure S41), demonstrating stealth capabilities comparable to the electromagnetic scattering levels of avian organisms [47]. Further, we calculated the EMW absorption performance at different incident angles of EMW. As shown in Figure 5i, Ni/HNC‐200 exhibited wide‐angle absorption, maintaining an EAB of more than 4.9 GHz within a 30° range. This remarkable angular‐robust RCS reduction, ultra‐high EMW absorption strength, highlights the practical feasibility of curvature engineering in the design of next‐generation EMW absorbers.

To assess practical applicability, Ni/HNC‐200 was blended into a flexible elastomer composite to fabricate shielding patches, which were then applied to communication‐chip surfaces for radiated field tests (Figure 5j; Figure S42). In the shielding test, covering the chip area of the mobile phone with the Ni/HNC‐200 EP significantly suppressed the electric field radiation of the device, reducing the average radiation level to less than one‑fifth of the original value. Specifically, the bare chip emitted a radiated field strength of 1,014.48 V/m, which the Ni/HNC‐200 EP reduced to 100.26 V/m, corresponding to a 90.1% reduction in emission intensity. These results validate that nanocurvature‐enhanced Ni‐N_4_ dipole polarization can be converted into effective radiation shielding for flexible shapes, demonstrating the real‐world potential to mitigate real‐world electromagnetic interference in consumer electronics such as chip packaging, wearable modules, and flexible devices.

## 3 Conclusion

This work establishes nanocurvature engineering as an effective strategy to activate dipolar polarization in atomically dispersed M‐N_4_ sites on hollow carbon spheres. By integrating click chemistry with template‐assisted synthesis, uniformly distributed Ni, Co, and Cu single atoms were anchored on curved carbon shells with precisely controlled diameters. Experimental results and density functional theory calculations collectively demonstrate that nanocurvature induces surface charge accumulation and electronic redistribution at M–N_4_ sites, enhancing dipole moments and polarizability and thereby strengthening intrinsic polarization loss. As a result, exceptional EMW absorption performance is achieved, exemplified by an ultralow minimum reflection loss of −74.1 dB for Ni/HNC‐200. Systematic simulations and flexible patch demonstrations further confirm the practical effectiveness of this approach. Overall, this study overcomes the symmetry‐imposed limitation of planar M–N_4_ coordination and defines nanocurvature as a geometry‐enabled design principle for next‐generation electromagnetic functional materials.

## 4 Materials and Methods

### 4.1 Materials

Nickel chloride hexahydrate (NiCl_2_·6H_2_O, ≥99.9%), copper chloride dehydrate (CuCl_2_·2H_2_O, ≥99.9%), cobalt chloride hexahydrate (CoCl_2_·6H_2_O, ≥99.9%), meso‐tetra(4phenyl) porphine (Por, ≥97%), trimethylamine (TEA, ≥99.5%) and pyrrole (≥99.9%) were purchased from Aladdin (Shanghai, China). Ammonium persulfate [(NH_4_)_2_S_2_O_8_, APS, ≥99.99%], thionyl chloride (SOCl_2_, ≥99.9%), 4‐dimethylaminopyridine (DMAP, ≥99.9%), and potassium hydroxide (KOH, ≥99.9%) were obtained from Macklin (Shanghai, China). Polystyrene microspheres were purchased by Jiangsu Xfnano Materials Tech Co., Ltd. All the chemicals were of analytical grade and used without further purification.

### 4.2 Synthesis of MPor (M: Ni, Cu, Co)

MPor was synthesized according to our previous work [6]. A solution containing 0.9 g of meso‐tetra(4‐phenyl)porphine and 3.3 g of nickel(II) chloride hexahydrate (NiCl_2_·6H_2_O) in 100 mL of N,N‐dimethylformamide (DMF) was prepared. The metallation reaction was conducted under reflux conditions at 140°C for 8 h with continuous stirring. After cooling, 150 mL of deionized water was added to the reaction mixture to induce precipitation. The resulting solid was isolated by vacuum filtration and sequentially washed three times with 1 m hydrochloric acid (HCl) to remove unreacted metal salts and impurities, followed by three washes with deionized water to neutralize residual acid. The purple solid was obtained by filtration and dried under vacuum for 24 h.

A portion of the dried purple solid (1.5 g) was dissolved in a mixed solvent system consisting of 50 mL tetrahydrofuran (THF) and 50 mL methanol. To this solution, 50 mL of an aqueous potassium hydroxide (KOH) solution (prepared by dispersing 5.26 g of KOH in deionized water) was added dropwise under stirring. The reaction mixture was heated to reflux at 85°C for 24 h to ensure complete demetalation and subsequent remetalation. After cooling to room temperature, sufficient deionized water was added to dissolve any residual solids. The solution was then acidified by the slow, dropwise addition of 1 m HCl until no further precipitate formation was observed. The resulting rufous solid (NiPor) was isolated by vacuum filtration, washed extensively with deionized water, and dried under vacuum.

Following an analogous procedure, copper(II) porphyrin (CuPor) and cobalt(II) porphyrin (CoPor) were synthesized by substituting NiCl_2_·6H_2_O with the corresponding metal salts (CuCl_2_·2H_2_O and CoCl_2_·6H_2_O, respectively).

### 4.3 Synthesis of Hollow HPPy Microspheres

The synthesis of hollow HPPy microspheres was adapted from a previously reported method with modifications. Polystyrene (PS) microspheres of varying diameters were selected as templates to achieve the desired core–shell dimensions.

In a typical procedure, 20 mL of an aqueous dispersion of PS microspheres (25 mg/mL) was ultrasonically dispersed in 1000 mL of ultrapure water for 3 h to ensure uniform distribution. Subsequently, 1 mL of freshly distilled pyrrole was dissolved in 20 mL of ethanol and added dropwise to the PS dispersion under continuous stirring. The chemical oxidative polymerization of pyrrole was initiated in an ice bath by sequentially adding 90 mL of hydrochloric acid (HCl, 37 wt.%) and 20.0 mL of an aqueous solution of ammonium persulfate (APS, 0.05 mg/mL) as the oxidant. The reaction mixture was stirred for 12 h to complete the polymerization process.

Following polymerization, the resulting core–shell structured product was thoroughly immersed in DMF to selectively remove the PS cores, yielding hollow polypyrrole microspheres. The sample was then washed repeatedly with deionized water to remove residual reagents and freeze‐dried to obtain the final HPPy material.

### 4.4 Synthesis of M/HNC (M: Ni, Cu, Co)

In general, MPor were covalently immobilized onto the surface of pre‐fabricated HPPy microspheres of varying sizes through an amidation reaction. Subsequent pyrolysis treatment yielded dimensionally tailored electromagnetic wave absorbers.

In a typical procedure, NiPor (300 mg) and SOCl_2_ (102 µL) were dissolved in 20 mL of DMF and sonicated for 15 min to facilitate the conversion of carboxyl groups to acyl chloride groups. Following the addition of DMAP (170 mg) as an amidation catalyst to form the feedstock solution. Separately, 100 mg of HPPy was charged into 30 mL of DMF, followed by dropwise addition of 173 µL of TEA and subsequent ultrasonication for a minimum of 60 min. Subsequently, the feedstock solution was added slowly to the above HPPy dispersion under continuous stirring. The reaction mixture was maintained at 80°C for 48 h to ensure complete amidation.

Following cooling to ambient temperature, the resulting black solid was collected by filtration and subsequently purified through six alternating washings with DMF and ethanol to remove residual reactants and impurities. The purified samples were dried under vacuum at 60°C for 24 h, yielding Ni/HPPy. The Ni/HPPy sample was subjected to pyrolysis at 700°C for 2 h under an argon atmosphere, with a controlled heating rate of 5°C/min. The final product was labeled as Ni/HNC.

Following an analogous procedure, Cu/HNC and Co/HNC were synthesized by substituting NiPor with CuPor and CoPor, respectively.

### 4.5 Material Characterization

The morphology and microstructure of the samples were characterized using a scanning electron microscope (SEM, Quant 250FEG) equipped with an energy‐dispersive spectrometer (EDS). Further microstructural analysis was performed by transmission electron microscopy (TEM) and high‐resolution TEM (HRTEM) using a JEM‐F200 field‐emission electron microscope. Single‐atom micrographs and energy‐dispersive X‐ray (EDX) elemental mappings were obtained using aberration‐corrected high‐angle annular dark‐field scanning transmission electron microscopy (AC HAADF‐STEM; FEI Themis Z). The crystal structure was analyzed by powder X‐ray diffraction (PXRD, Bruker D8 Discover). Fourier‐transform infrared spectroscopy (FTIR) was performed on a NICOLET IS 20 spectrograph. Raman spectra were collected using a Raman spectrophotometer (Aramis) with a 532 nm solid‐state laser. The chemical states and chemical bonds were analyzed by X‐ray photoelectron spectroscopy (XPS, PHI QUANTERA II), with the data calibrated using the C 1s peak at 284.8 eV. Inductively coupled plasma optical emission spectroscopy (ICP‐OES) was performed on a PerkinElmer 8300. Conductivity measurements were obtained using a four‐point probe (RTS‐8). Electrochemical impedance spectroscopy (EIS) was conducted using an electrochemical workstation (CHI 660E) with a three‐electrode configuration, including a saturated Ag/AgCl reference electrode, a Pt wire counter electrode, and a 1 m Na_2_SO_4_ electrolyte.

### 4.6 X‐Ray Absorption Fine Structure measurement

Ni K‐edge X‐ray absorption fine structure (XAFS) measurements were performed at the BL14W beamline of the Shanghai Synchrotron Radiation Facility (SSRF), China. Samples were prepared by mounting them in aluminum holders and sealing with Kapton tape prior to analysis. The XAFS spectra were collected using a Bruker 5040 4‐channel Silicon Drift Detector (SDD), in fluorescence modes. The Ni K‐edge X‐ray absorption near‐edge structure (XANES) spectra showed consistent line shapes and peak positions across repeated scans for each sample. Transmission mode was used to acquire the reference spectra for Ni‐foil, NiPc, and NiO. Data processing and analysis were carried out using the Athena software package based on the IFEFFIT program to determine the structural environment of Ni atoms. The first shell coordination belongs to Ni‐N. The best‐fitting results were obtained to meet the statistical criteria R < 0.02.

### 4.7 DFT Calculation Method

All calculations were based on density functional theory (DFT) as implemented in the Vienna Ab initio Simulation Package (VASP) [48]. We employed the projector augmented‐wave (PAW) method to describe the electron‐ion interactions [49]. The Perdew‐Burke‐Ernzerhof (PBE) parameterization of the generalized gradient approximation (GGA) was employed to describe the exchange‐correlation functional [50, 51].

A plane‐wave basis set with a kinetic energy cutoff of 400 eV was used for the expansion of the wave functions. The initial calculations were performed using a periodic graphite model with a (7 × 3) supercell. The Brillouin zone was sampled using a Monkhorst‐Pack k‐point mesh of 2 × 2 × 1 for structural relaxations. The electronic self‐consistent cycle was considered converged when the total energy change between successive steps was less than 1 × 10^−7^ eV. Geometry optimization was performed until the Hellmann‐Feynman forces on all atoms were below 0.02 eV/Å.

Polarizability calculations were conducted using the Gaussian 16 software package. Wavefunction analyses were performed with Multiwfn [52]. The electronic structure for these calculations was obtained at the PBE0/aug‐cc‐pVDZ level of theory [53]. The aug‐cc‐pVDZ basis set includes diffuse functions, which are essential for accurately achieve the calculation of polarizability.

## Author Contributions

Conceptualization: A.X. and S.C.; Methodology: A.X., W.L., and S.C.; Investigation: D.S., S.C., J.Z., M.Z., and B.Z.; Visualization: D.S. and S.C.; Supervision: A.X., W.L., and S.C.; Writing – original draft: D.S. and S.C.; Writing – review & editing: A.X., W.L., S.C., and D.S.

## Conflicts of Interest

The authors declare no conflicts of interest.

## Supporting information




**Supporting File 1**: advs75115‐sup‐0001‐MovieS1.mp4.

**Supporting File 2**: advs75115‐sup‐0002‐MovieS2.mp4.

**Supporting File 3**: advs75115‐sup‐0003‐MovieS3.mp4.

**Supporting File 4**: advs75115‐sup‐0004‐MovieS4.mp4.

**Supporting File 5**: advs75115‐sup‐0005‐SuppMat.docx.

## Data Availability

All data needed to evaluate the conclusions in the paper are present in the paper and/or the Supplementary Materials. Any additional requests for information can be directed to and will be fulfilled by the corresponding authors.
