# Supplementary material for: Nanocurvature‐Activated Dipolar Polarization in M–N4 Single‐Atom Sites for High‐Performance Electromagnetic Wave Absorption
Source: Adv Sci (Weinh). 2026 Mar 31;13(34):e75115. doi: 10.1002/advs.75115 (PMC13285111; doi:10.1002/advs.75115)
Supplement: Supplementary file 5 — Supporting File 5: advs75115‐sup‐0005‐SuppMat.docx. [file ADVS-13-e75115-s004.docx]

**Supporting Information**

**Nanocurvature-Activated Dipolar Polarization in M–N_4_ Single-Atom Sites for High-Performance Electromagnetic Wave Absorption**

*Daohu Sheng, Siyao Cheng*, Mengmeng Zhang, Jinglei Zhang, Xufei Zhu, Weijin Li*, Bo Zhang, Aming Xie**

D. Sheng, S. Cheng, M. Zhang, A. Xie

School of Safety Science and Engineering, Nanjing University of Science and Technology, Nanjing 210094, P. R. China.

E-mail: chengsiyao@fjirsm.ac.cn; xieaming@njust.edu.cn

D. Sheng, J. Zhang, X. Zhu

School of Chemistry and Chemical Engineering, Nanjing University of Science and Technology, Nanjing 210094, P. R. China.

W. Li

MIIT Key Laboratory of Advanced Display Materials and Devices & Materials Physical and Chemical Research and Practice Center, College of Materials Science and Engineering, Nanjing University of Science and Technology, Nanjing, China.

E-mail: wjli@njust.edu.cn

B. Zhang

CAS Key Laboratory of Science and Technology on Applied Catalysis, Dalian Institute of Chemical Physics, Chinese Academy of Sciences, Dalian 116023, China.

S. Cheng

State Key Laboratory of Structural Chemistry Fujian Institute of Research on the Structure of Matter, Chinese Academy of Sciences, Fuzhou 350002, P. R. China.

Supplementary Text

**Electromagnetic performance**

According to transmission line theory, the EM parameters of the sample are measured using the coaxial method. Prior to experimentation, each sample, constituting 9 wt.%, was compacted into annular forms with an inner diameter (Φ_in_) of 3.04 mm and an outer diameter (Φ_out_) of 7.00 mm using paraffin as a binding agent. The efficacy of electromagnetic absorption was subsequently calculated through the application of the ensuing mathematical formulations^[[1]](#endnote-1)^.

$$\begin{aligned} \text{ }\text{RL}\left( \text{dB} \right)\text{=20}\text{log}_{\text{10}}\frac{\left| \text{z}_{\text{in}}\text{-}\text{z}_{\text{0}} \right|}{\left| \text{z}_{\text{in}}\text{+}\text{z}_{\text{0}} \right|}\text{ } \#\text{(S1)} \end{aligned}$$

$$\begin{aligned} \text{z}_{\text{in}}\text{=}\text{z}_{\text{0}}\sqrt{\frac{\text{μ}_{\text{r}}}{\text{ε}_{\text{r}}}}\text{tanh}\left( \frac{\text{2π}\text{jfd}}{\text{c}}\sqrt{\text{μ}_{\text{r}}\text{ε}_{\text{r}}} \right)\text{ } \#\text{(S2)} \end{aligned}$$

where Z_in_ denotes the normalized input impedance of the absorbers, Z_0_ stands for the impedance of free space, f corresponds to the frequency of the EMW, d is the thickness of the absorbers and c denotes the velocity of EMW propagation in a vacuum.

Polarization relaxation is evaluated through Cole-Cole semicircles. Following Debye relaxation theory, the related equations of ε′ and ε″ are as follows^[[2]](#endnote-2)^:

$$\begin{aligned} \text{ε}^{'}\text{=}\text{ε}_{\text{∞}}\text{+}\frac{\text{ε}_{\text{s}}\text{-}\text{ε}_{\text{∞}}}{\text{1+}\text{ω}^{\text{2}}\text{ τ}^{\text{2}}} \#\text{(}\text{S}\text{3)} \end{aligned}$$

$$\begin{aligned} \text{ε}^{''}\text{=}\text{ε}_{\text{p}}^{''}\text{+}\text{ε}_{\text{c}}^{''}\text{=ωτ}\frac{\text{ε}_{\text{s}}\text{-}\text{ε}_{\text{∞}}}{\text{1+}\text{ω}^{\text{2}}\text{τ}^{\text{2}}}\text{+}\frac{\text{σ}}{\text{ω}\text{ε}_{\text{0}}} \#\text{(}\text{S}\text{4)} \end{aligned}$$

$$\begin{aligned} \left( \text{ε}^{'}\text{-}\frac{\text{ε}_{\text{s}}\text{+}\text{ε}_{\text{∞}}}{\text{2}} \right)^{\text{2}}\text{+}\left( \text{ε}^{''} \right)^{\text{2}}\text{=}\left( \frac{\text{ε}_{\text{s}}\text{-}\text{ε}_{\text{∞}}}{\text{2}} \right)^{\text{2}}\text{ }\text{ }\text{ } \#\text{(}\text{S}\text{5)} \end{aligned}$$

where Z_in_ denotes the normalized input impedance of the absorbers, Z_0_ stands for the impedance of free space, f corresponds to the frequency of the EMW, d is the thickness of the absorbers and c denotes the velocity of EMW propagation in a vacuum.

The attenuation ability can be revealed by the attenuation constant (α), which can be calculated via following equations^[[3]](#endnote-3)^:

$$\begin{aligned} \text{ α=}\frac{\sqrt{\text{2}}\text{πf}}{\text{c}}\text{×}\sqrt{\left( \text{μ}^{''}\text{ε}^{''}\text{-}\text{μ}^{'}\text{ε}^{'} \right)\text{+}\sqrt{\left( \text{μ}^{''}\text{ε}^{''}\text{-}\text{μ}^{'}\text{ε}^{'} \right)^{\text{2}}\text{+}\left( \text{μ}^{'}\text{ε}^{''}\text{+}\text{μ}^{''}\text{ε}^{'} \right)^{\text{2}}}} \#\text{(}\text{S}\text{6)} \end{aligned}$$

**Non-linear least squares Fitting**

Conductive loss (ε_c_״) and polarization loss (ε_p_״) values were fitted to each compliance using Non-linear least squares via running Python. Non-linear least squares fitting is a classic and precise way to fit a curve. According to Debye theory, the model function in this work as follows ^[[4]](#endnote-4)^:

$$\begin{aligned} \text{ }\text{ε=}\text{ε}_{\text{∞ }}\text{+}\frac{\text{(ε}_{\text{s}}\text{-}\text{ε}_{\text{∞}}\text{)}}{\text{(1+}\text{ω}^{\text{2}}\text{τ}^{\text{2}}\text{)}}\text{-}\text{i}\left[ \frac{\left( \text{ε}_{\text{s}}\text{-}\text{ε}_{\text{∞}} \right)\text{ωτ}}{\left( \text{1+}\text{ω}^{\text{2}}\text{τ}^{\text{2}} \right)}\text{+}\frac{\text{σ}}{\left( \text{ω}\text{ε}_{\text{0}} \right)} \right] \#\text{(}\text{S}\text{7)} \end{aligned}$$

Among them,

$$\text{ε}^{'}\text{=}\text{ε}_{\text{∞ }}\text{+}\frac{\text{(ε}_{\text{s}}\text{-}\text{ε}_{\text{∞}}\text{)}}{\text{(1+}\text{ω}^{\text{2}}\text{τ}^{\text{2}}\text{)}}$$

$$\text{ε}^{''}\text{=}\text{ε}_{\text{p}}^{''}\text{+}\text{ε}_{\text{c}}^{''}\text{=}\frac{\left( \text{ε}_{\text{s}}\text{-}\text{ε}_{\text{∞}} \right)\text{ωτ}}{\text{(1+}\text{ω}^{\text{2}}\text{τ}^{\text{2}}\text{)}}\text{+}\frac{\text{σ}}{\text{(ω}\text{ε}_{\text{0}}\text{)}}$$

where $\varepsilon_{\infty}$ is optical dielectric constant, $\varepsilon_{s}$is static dielectric constant, $\varepsilon_{0}$ is free space dielectric constant, τ is the relaxation time, and σ is the conductivity. The parameters needed to fitted are $\text{ε}_{\text{∞}}$, $\text{ε}_{\text{s}}$, $\text{ε}_{\text{0}}$, τ and σ, which are signed as a group β. The sum of squares is $\text{S=}\sum_{\text{i}\text{=1}}^{\text{m}} \text{r}_{\text{i}}^{\text{2}}$, where $\text{r}_{\text{i}}\text{=}\text{ε}_{\text{fit}}\text{-ε}$. Then our goal is minimizing the S by adjusting β. The mean number is our result.

**Electromagnetic radiation detection experiment**

25 wt% Ni/HNC-200 and 80 wt% poly(vinylidene fluoride)-hexafluoropropylene were stirred uniformly with DNF as a dispersant and poured into a mold. After the DMF was air-dried naturally, a Ni/HNC-200 electronic patch (Ni/HNC-200 EP) with a thickness of 2.83 mm was formed. The EP was then applied to cover the surface of a mobile phone circuit. The dynamic radiation values were measured using an electromagnetic radiation detector.

**RCS simulation with CST Studio Suite**

The RCS values of M/HNC-200 (M = Ni, Cu, Co) were investigated using the CST Microwave Studio to study the far-field applications within the frequency band of 2-18 GHz. In this simulation, use a metal back plate with dimensions of 300 × 300 mm^2^ and material settings of perfect electrical conductor (PEC) as the simulation model. Moreover, the surface of PEC was coated with a layer of absorber. The model was in the XOY plane. The boundary conditions were applied with the electric field along x direction and the magnetic field along y direction. The open (add space) boundary conditions were used in all directions. The scattering directions were set as ≈ 0–360° for θ, and 0–360° for ϕ. The RCS values is expressed by the following equation:

$$\begin{aligned} \text{ }\text{σ}\left( \text{dB}\text{m}^{\text{2}} \right)\text{=10}\log\left( \frac{\text{4πS}}{\text{λ}^{\text{2}}}\left| \frac{\text{E}_{\text{s}}}{\text{E}_{\text{i}}} \right| \right)^{\text{2}} \#\text{(}\text{S}\text{8)} \end{aligned}$$

where *S* and *λ* represent the area of the simulation plate and the wavelength of the EMW, respectively. E_s_ and E_i_ represent the electric field intensity of transmitting waves and the electric field intensity of the receiving waves, respectively.

**Note S1 Nanocurvature Affects the Distribution of Surface Charges**

**
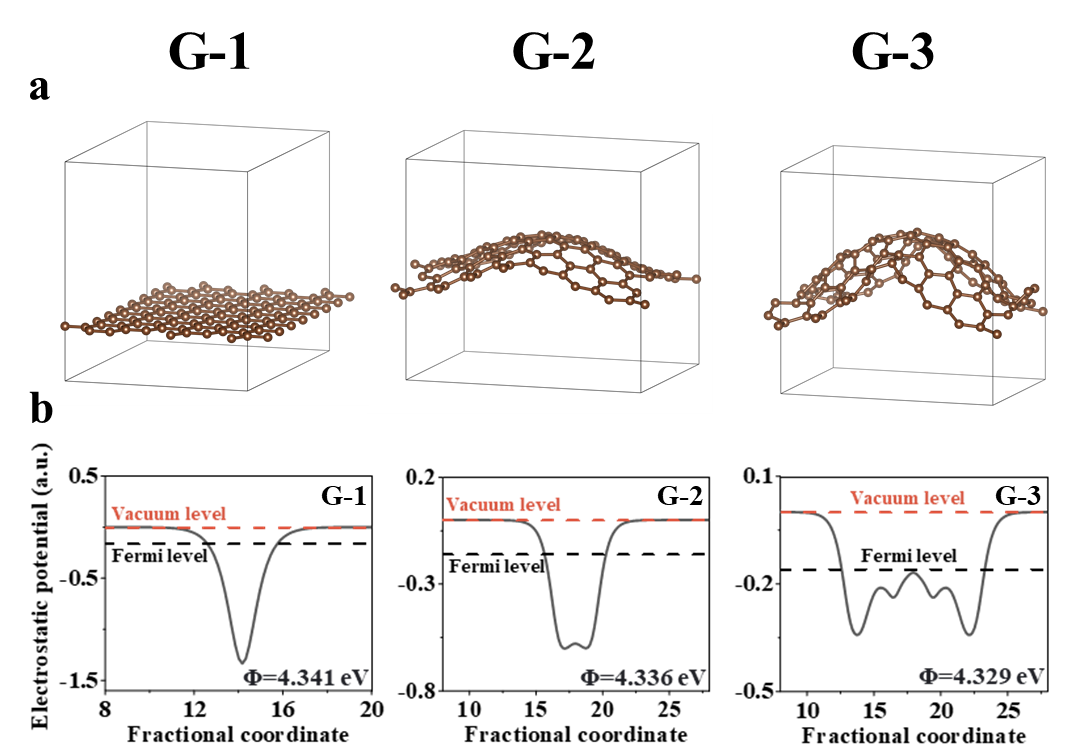
**

Figure S1. (a) Computational models of graphene with different nanocurvatures (From left to right, they are G-1, G-2, G-3, the curvature of graphene progressively increases). (b) Work function of G-1, G-2 and G-3.


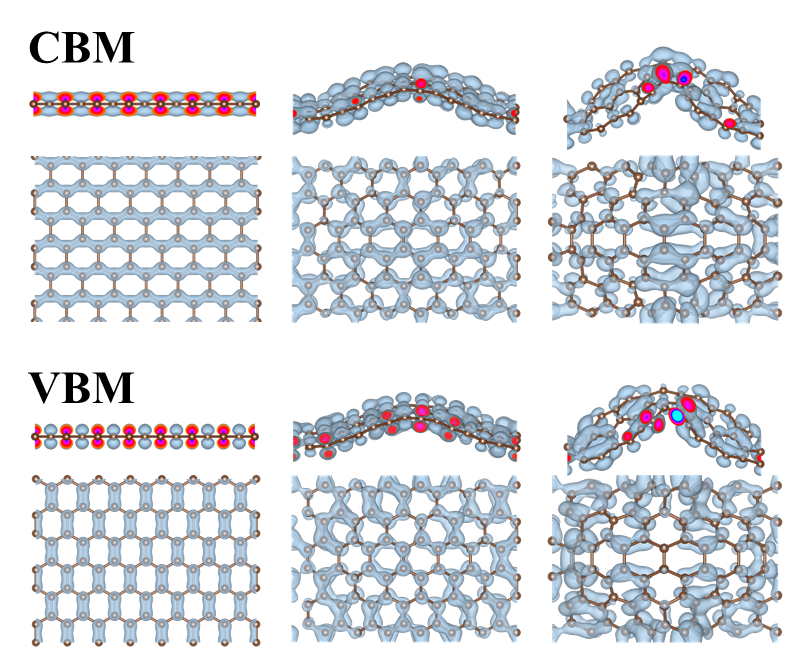


Figure S2. The calculated charge density of frontier molecular orbitals of graphene with various nanocurvature.


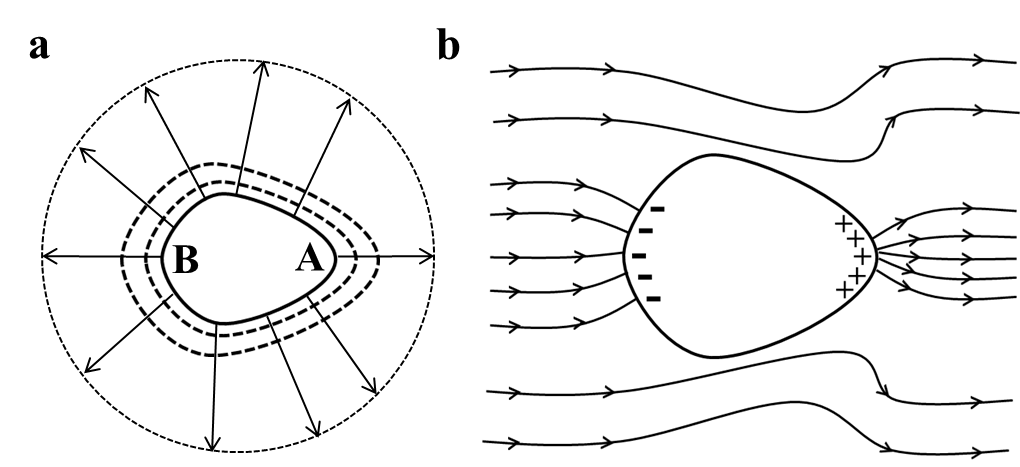


Figure S3. Curvature versus surface charge distribution. (a) Isopotential surface of anoncent rosymmetric conductor, and (b) schematic of the charge distribution of a non-centrosymmetric conductor in an electromagnetic field.

**Note S2 Absorber Design and Phase Structure Characterization**

This study successfully covalently grafted carboxyl-functionalized nickel porphyrin onto hollow polypyrrole nanospheres of varying diameters using a template-assisted approach and click-limited strategy. Following carbonization, a series of Ni/HNC-X EMW absorbers featuring atomically dispersed nickel sites were prepared.

All samples with different diameters successfully formed single atomic dispersion sites with a local structure of Ni-N₄, without the formation of nickel nanoparticles or clusters. Notably, despite variations in the nanocurvature (diameter) of the hollow carbon spheres, the local coordination environment of Ni single atoms (Ni-N₄), loading (approximately 0.1 wt%), and graphitization degree of the carbon matrix remained consistent and unaffected by particle size changes. These results demonstrate that the adopted synthesis strategy effectively suppresses metal agglomeration during high-temperature pyrolysis, thereby creating highly consistent single-metal sites on carriers with different curvatures. This provides an ideal model system for investigating the effects of nanocurvature.


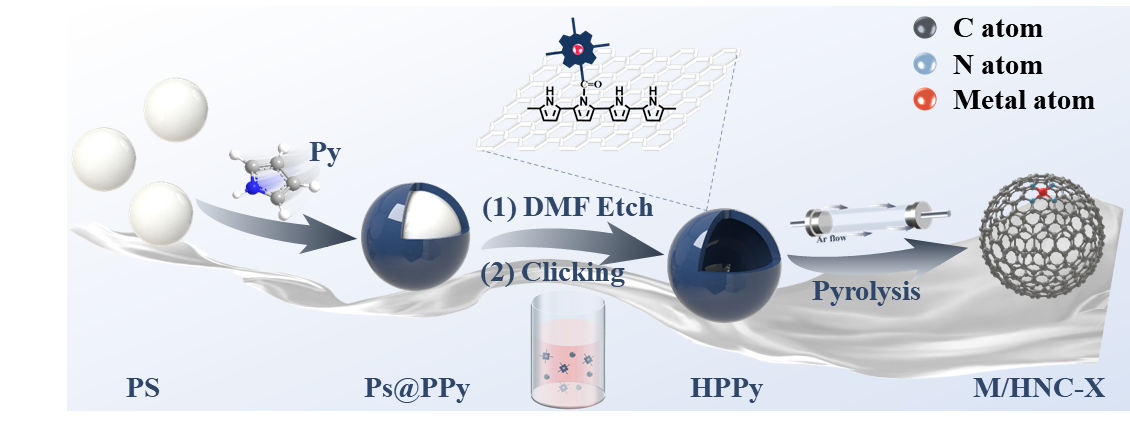


Figure S4. Schematic of the synthesis of M/HNC-X (M = Ni, Cu, Co, X= 50, 200, 580, 970).

Figure S5. FT-IR spectrum of NiPor/HPPy-X (X = 50, 200, 580, 970). Amido group can be identified in the NiPor/HPPy-X to indicate successful grafting of NiPor unit onto the substrate via click reaction.


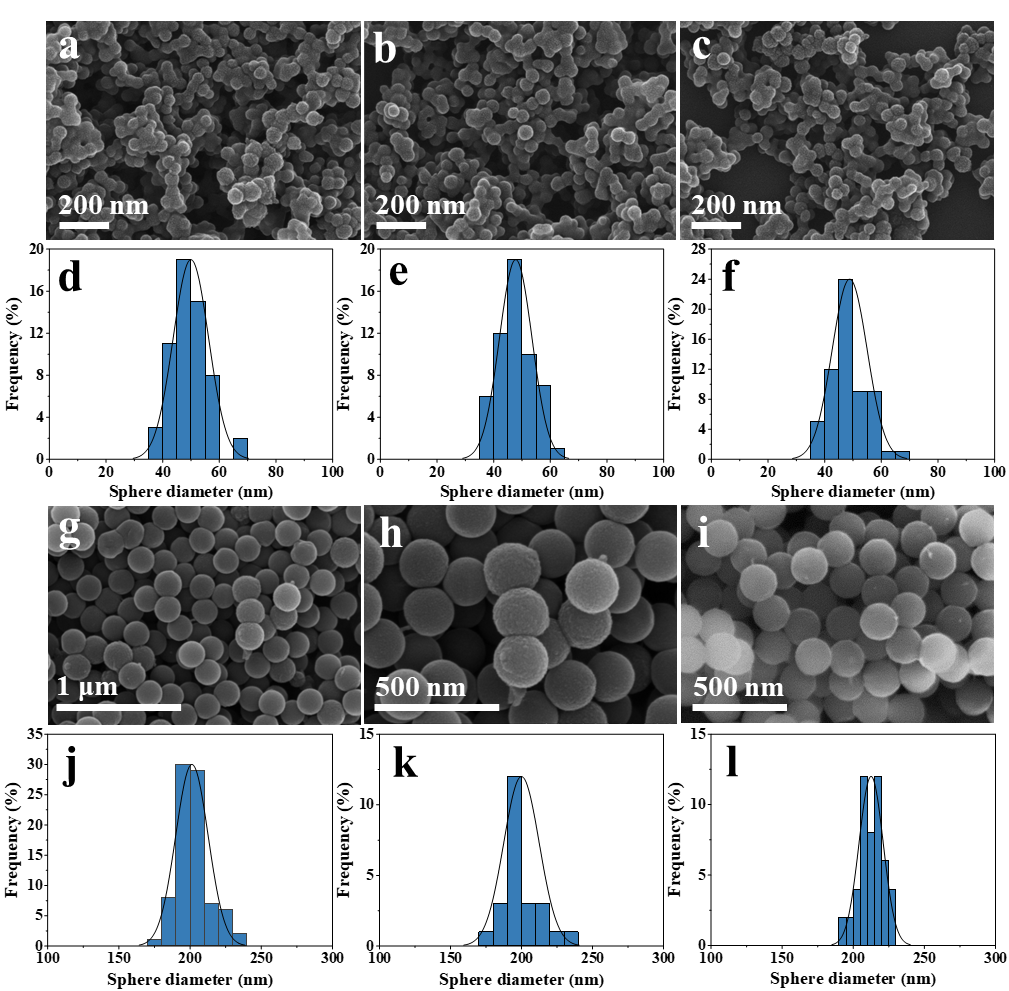


Figure S6. SEM images of (a, b, c) Ni/HNC-50 and (g, h, i) Ni/HNC-200. Size distribution analysis of (d, e, f) Ni/HNC-50 and (j, k, l) Ni/HNC-200.


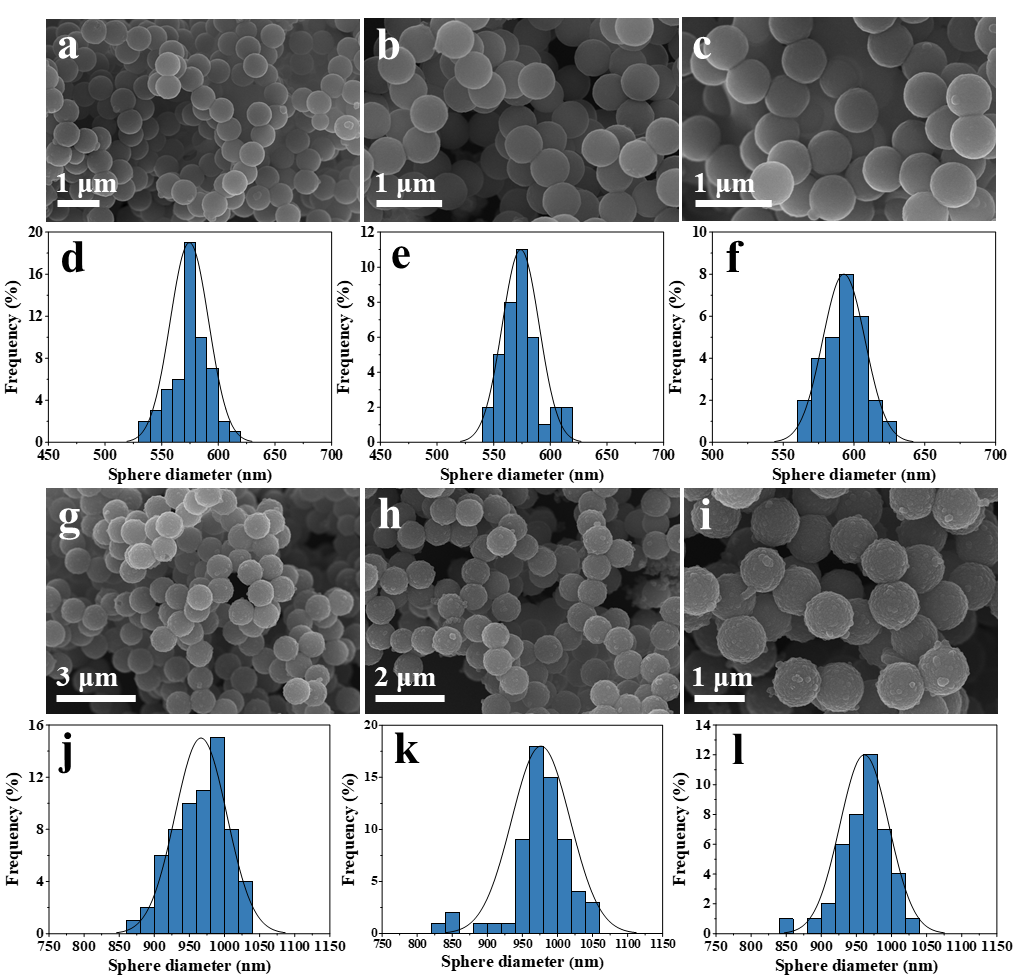


Figure S7. SEM images of (a, b, c) Ni/HNC-580 and (g, h, i) Ni/HNC-970. Size distribution analysis of (d, e, f) Ni/HNC-580 and (j, k, l) Ni/HNC-970.


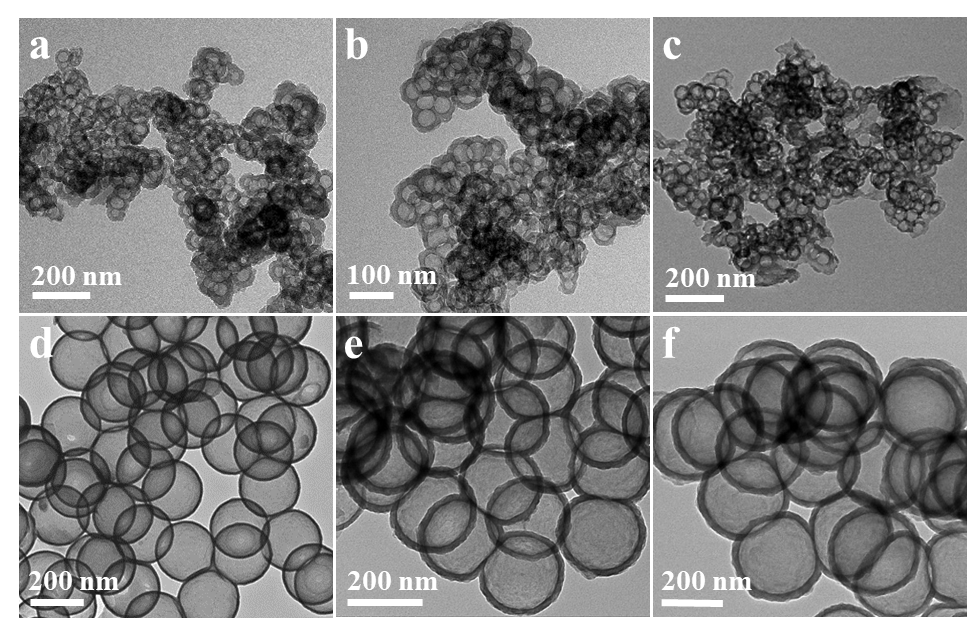


Figure S8. TEM images of (a, b, c) Ni/HNC-50 and (d, e, f) Ni/HNC-200.


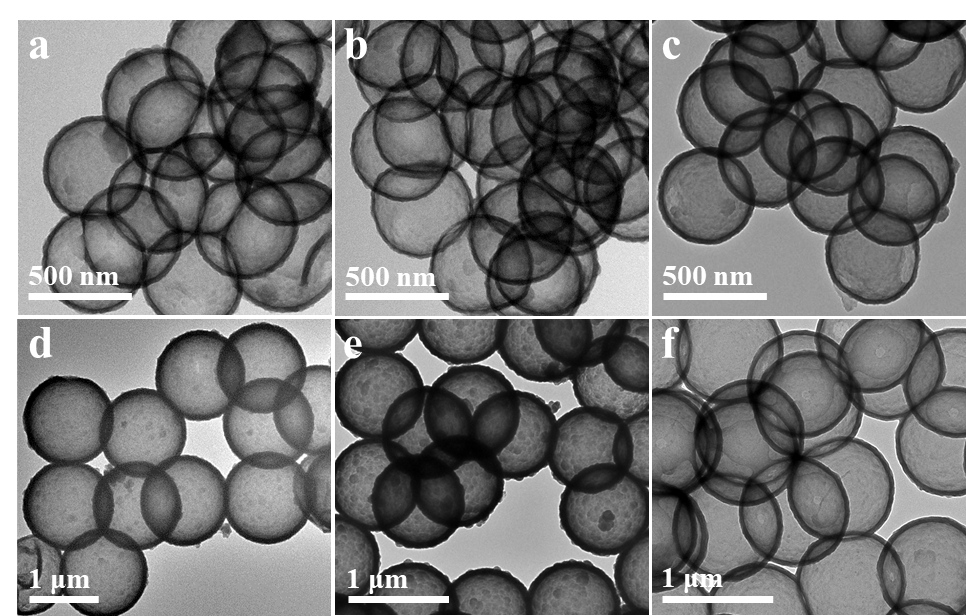


Figure S9. TEM images of (a, b, c) Ni/HNC-580 and (d, e, f) Ni/HNC-970.

Figure S10. XRD patterns of HNC and Ni/HNC-X (X = 50, 200, 580, 970).


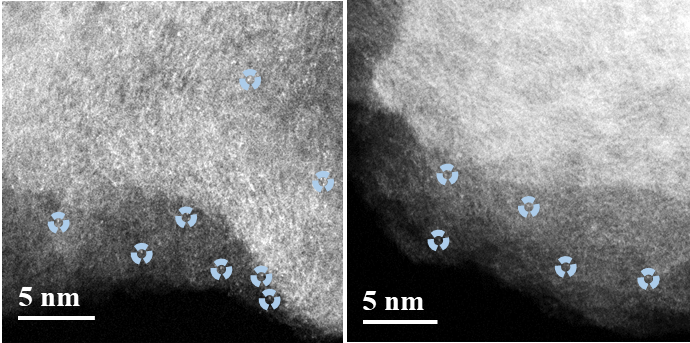


Figure S11. AC-HAADF-STEM images of Ni/HNC-200.


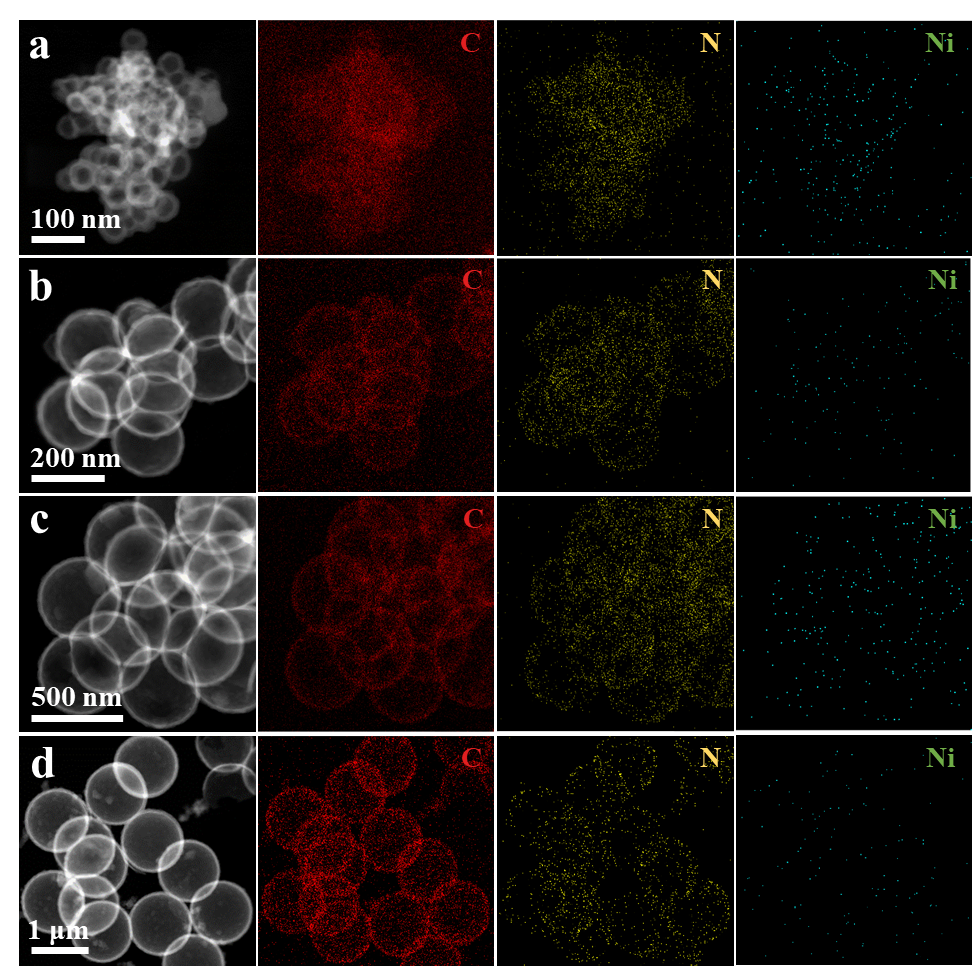


Figure S12. HAADF-STEM and corresponding EDS mapping images of (a) Ni/HNC-50, (b) Ni/HNC-200, (c) Ni/HNC-580, and (d) Ni/HNC-970.

Figure S13. ICP-OES spectrum of Ni/HNC-X (X = 50, 200, 580, 970).

Figure S14. Raman patterns of Ni/HNC-X (X = 50, 200, 580, 970).


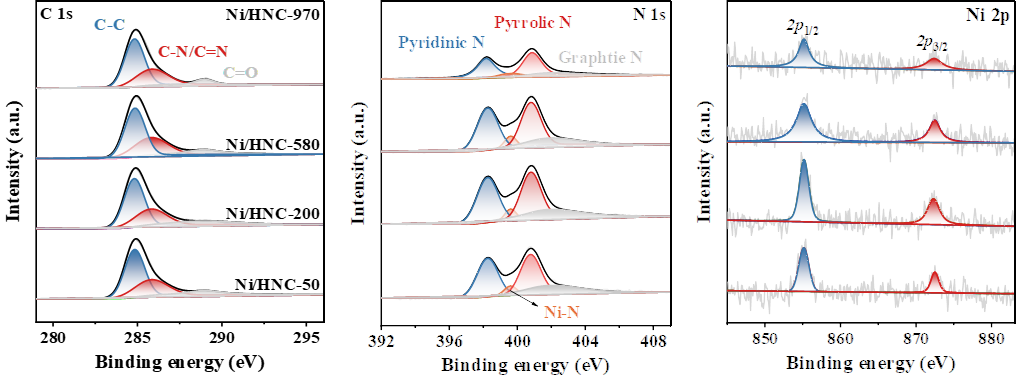


Figure S15. XPS analysis of Ni/HNC-X (X = 50, 200, 580, 970).

Figure S16. Calculated ratios of the different N species.

**Note S3 Effect of Nanocurvature on Dielectric Properties**

Through systematic measurement and analysis of the electromagnetic parameters of Ni/HNC-X samples with varying curvatures, all samples exhibited an absorption mechanism dominated by dielectric loss, with magnetic loss being negligible. Moreover, the dielectric loss capacity significantly increased with rising nanocurvature. EPR spectroscopy and electrochemical impedance spectroscopy further demonstrate that nanocurvature effectively enhances the polarization capability of Ni-N₄ dipoles under alternating electromagnetic fields by increasing unpaired electron density and improving charge mobility, thereby fundamentally improving the dielectric loss performance of the absorber.


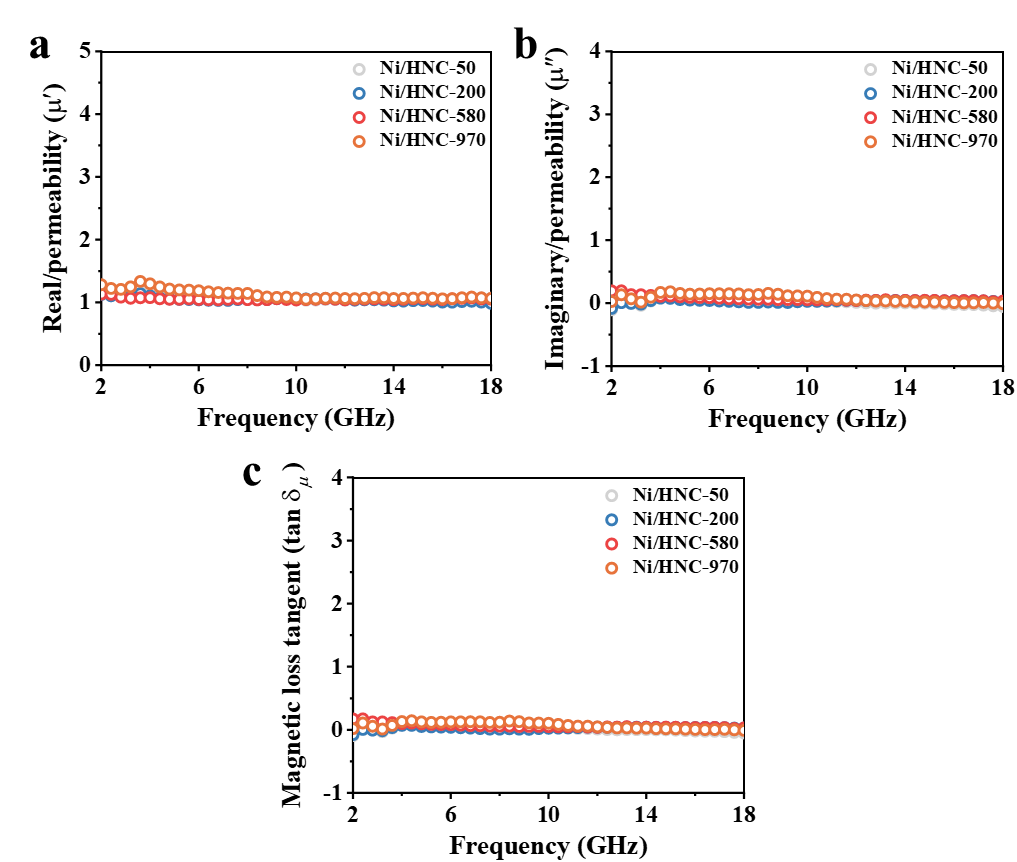


Figure S17. (a) Real and (b) imaginary part of complex permeability. (c) Magnetic loss angular tangent of the samples Ni/HNC-X (X = 50, 200, 580, 970).


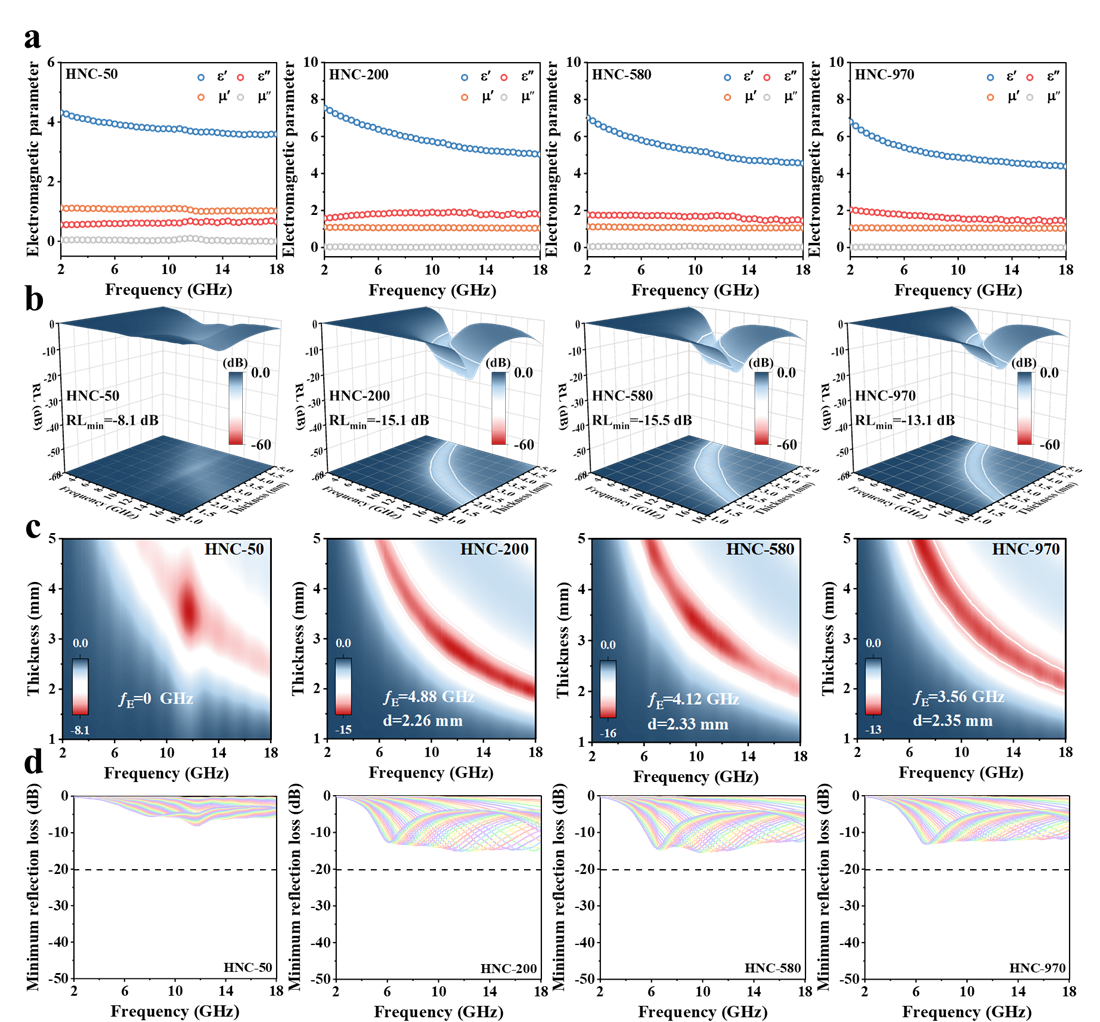


Figure S18. (a) Electromagnetic parameters, (b) 3D RL plots, (c) 2D RL plots and (d) RL curves (matching thickness: 1-5 mm; Increment: 0.1 mm) of HNC-X (X = 50, 200, 580, 970).


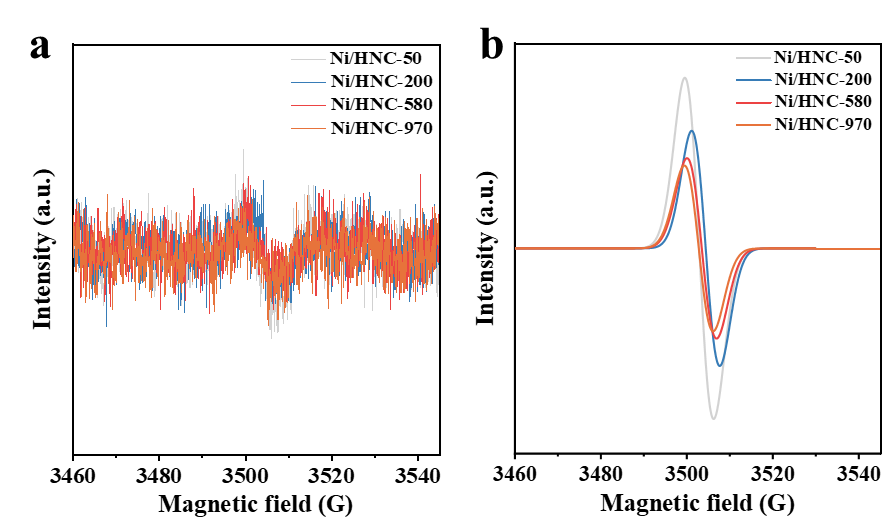


Figure S19. (a) EPR pattern and (b) EPR pattern data fitting of Ni/HNC-X (X = 50, 200, 580, 970).

Figure S20. Electrochemical impedance spectra of Ni/HNC-X (X = 50, 200, 580, 970).

**Note S4 Ni-N_4_ Dipole Polarization Enhancement Mechanism**

Differential charge results indicate that increased nanocurvature promotes electron transfer from Ni atoms to N atoms, leading to non-uniform spatial charge distribution at Ni-N₄ sites and thereby enhancing local electrostatic polarization. Furthermore, Hirshfeld population analysis confirms that Ni and N atoms acquire increased valence electron density with rising curvature. This effect intensifies charge localization and spatial inhomogeneity, enabling more electrons to participate in dipole relaxation under alternating electromagnetic fields. Consequently, the intrinsic polarization loss characteristics of each Ni–N₄ dipole are optimized, significantly improving the dielectric loss of high-curvature samples.


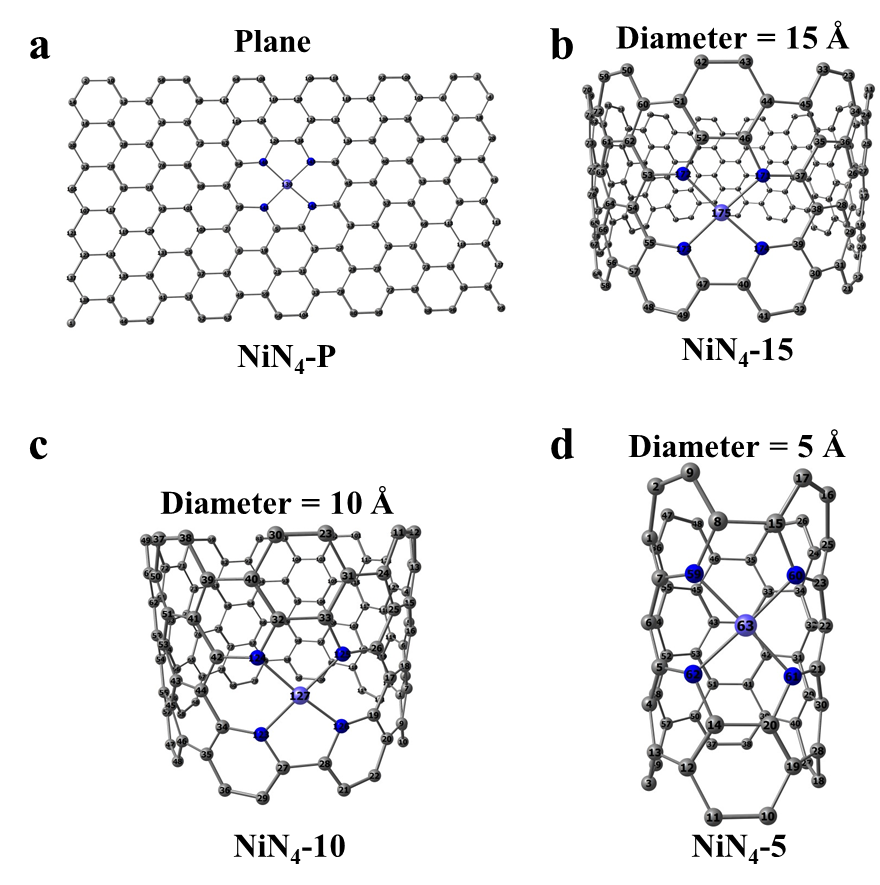


Figure S21. Graphene-based Ni-N₄ models with increasing curvature (P represents plane; 5, 10, 15 represent model diameter in Å).


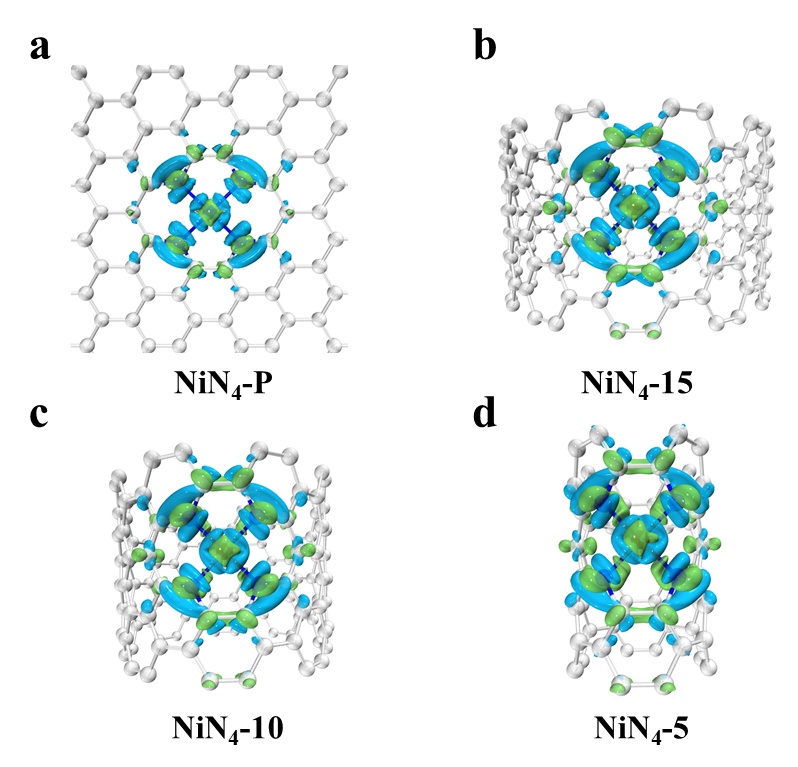


Figure S22. Differential charge densities plots of (a) NiN_4_-P, (b) NiN_4_-15, (c) NiN_4_-10 and (d) NiN_4_-5.


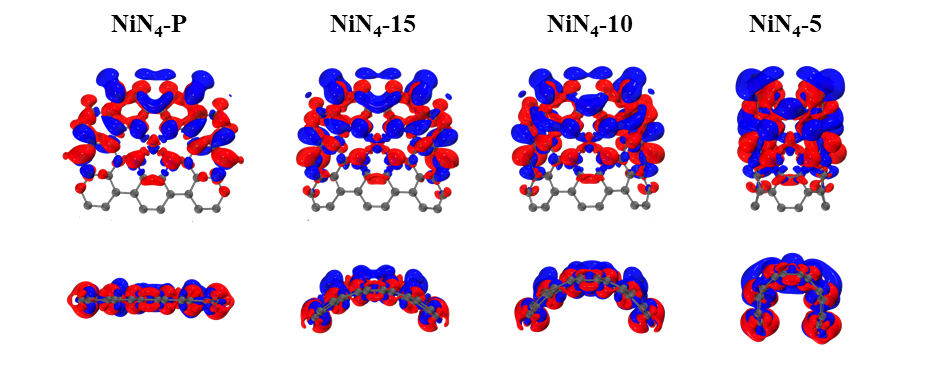


Figure S23. Hyperpolarizability densities of NiN_4_-P, NiN_4_-15, NiN_4_-10 and NiN_4_-5 (Red and blue regions representing positive and negative spatial contributions, respectively).


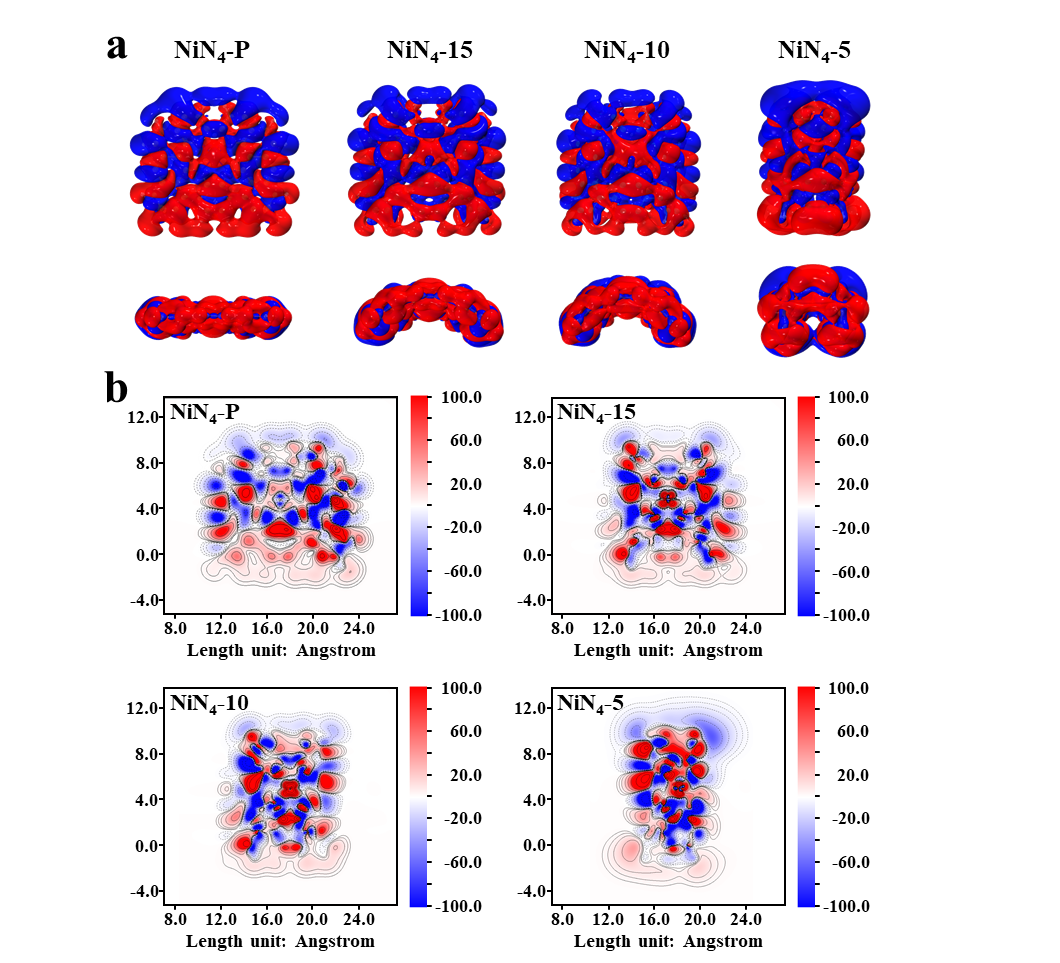


Figure S24. (a) Second-order hyperpolarizability densities of NiN_4_-P, NiN_4_-15, NiN_4_-10 and NiN_4_-5. (b) Color-filled contour maps of x-component of the hyperpolarizability of NiN_4_-P, NiN_4_-15, NiN_4_-10 and NiN_4_-5 in static electric field (Red and blue regions representing positive and negative spatial contributions, respectively).


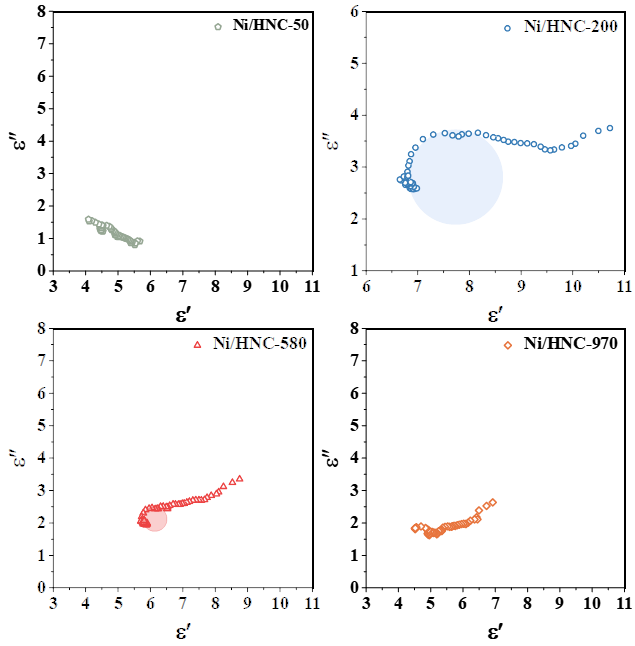


Figure S25. Cole-cole semicircles of Ni/HNC-X (X = 50, 200, 580, 970).

Figure S26. The calculated ε_c_″ and ε_p_″ (Data obtained by Python fitting).

**Note S5** **Verification of the Generality of Design Principles**

In the section, to validate the generality of nanocurvature-induced electronic effects on EMW absorption enhancement, we synthesized analogs with Cu and Co metal centers. These exhibited similar performance trends and significantly improved absorption rates. Among them, Cu/HNC-200 and Co/HNC-200 achieved RLmin values of −76.3 dB at 8.96 GHz and −63.3 dB at 6.52 GHz, respectively, supporting the proposed nanocurvature-driven enhancement.


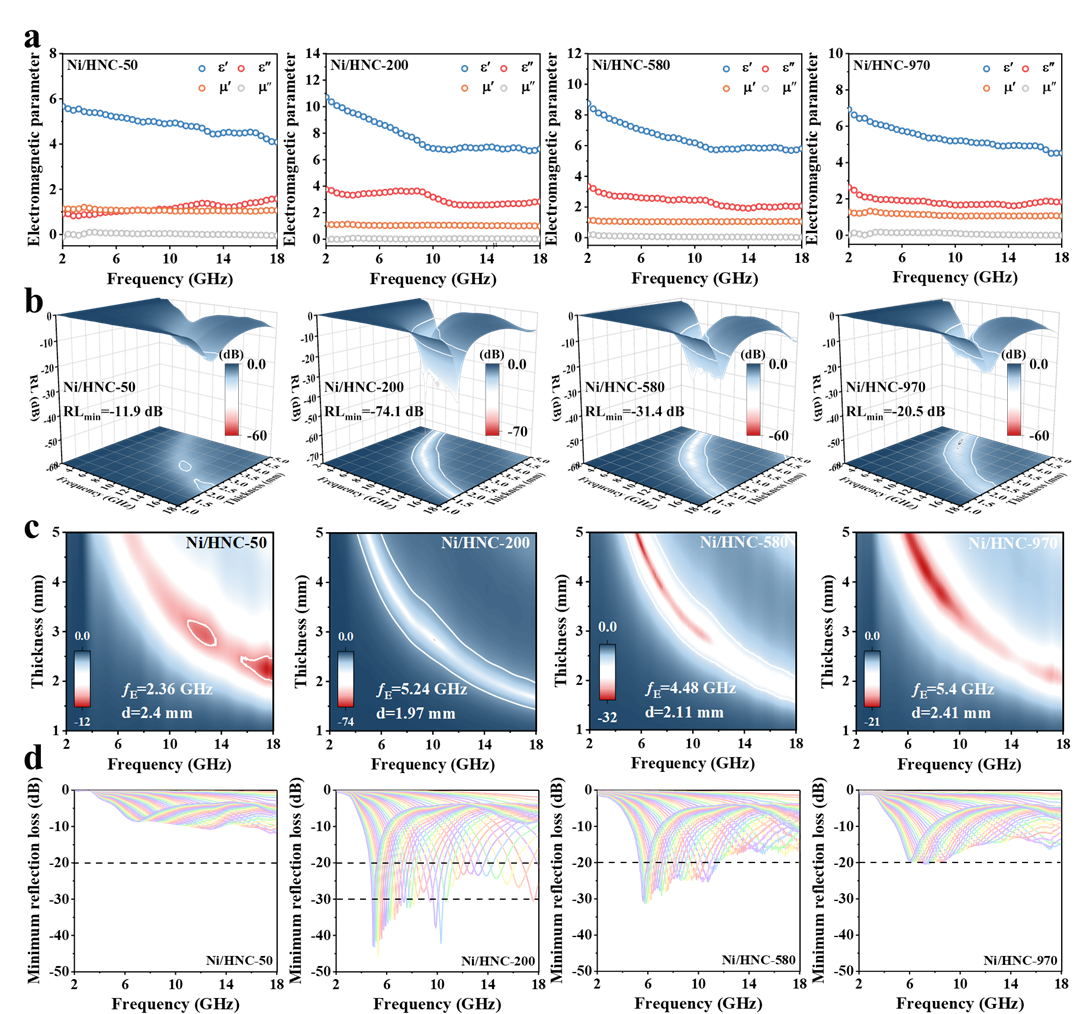


Figure S27. (a) Electromagnetic parameters, (b) 3D RL plots, (c) 2D RL plots and (d) RL curves (matching thickness: 1-5 mm; Increment: 0.1 mm) of Ni/HNC-X (X = 50, 200, 580, 970).


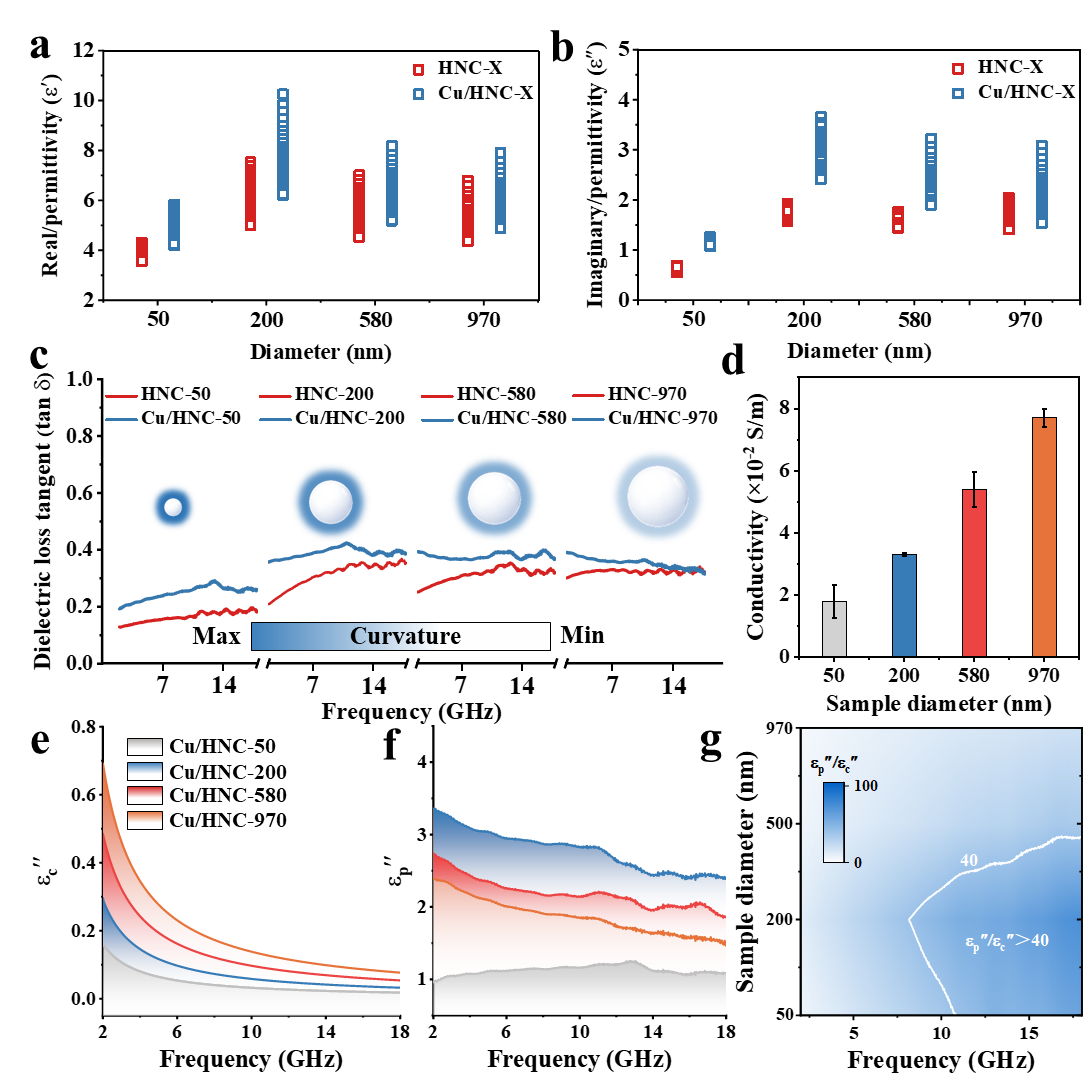


Figure S28. (a) Real and (b) imaginary part of complex permittivity. (c) Dielectric loss tangent, (d) electrical conductivity of samples dispersed in paraffin wax, (e) conduction loss, (f) polarization loss, and (g) polarization loss/conduction loss ratios of Cu/HNC-X (X = 50, 200, 580, 970).


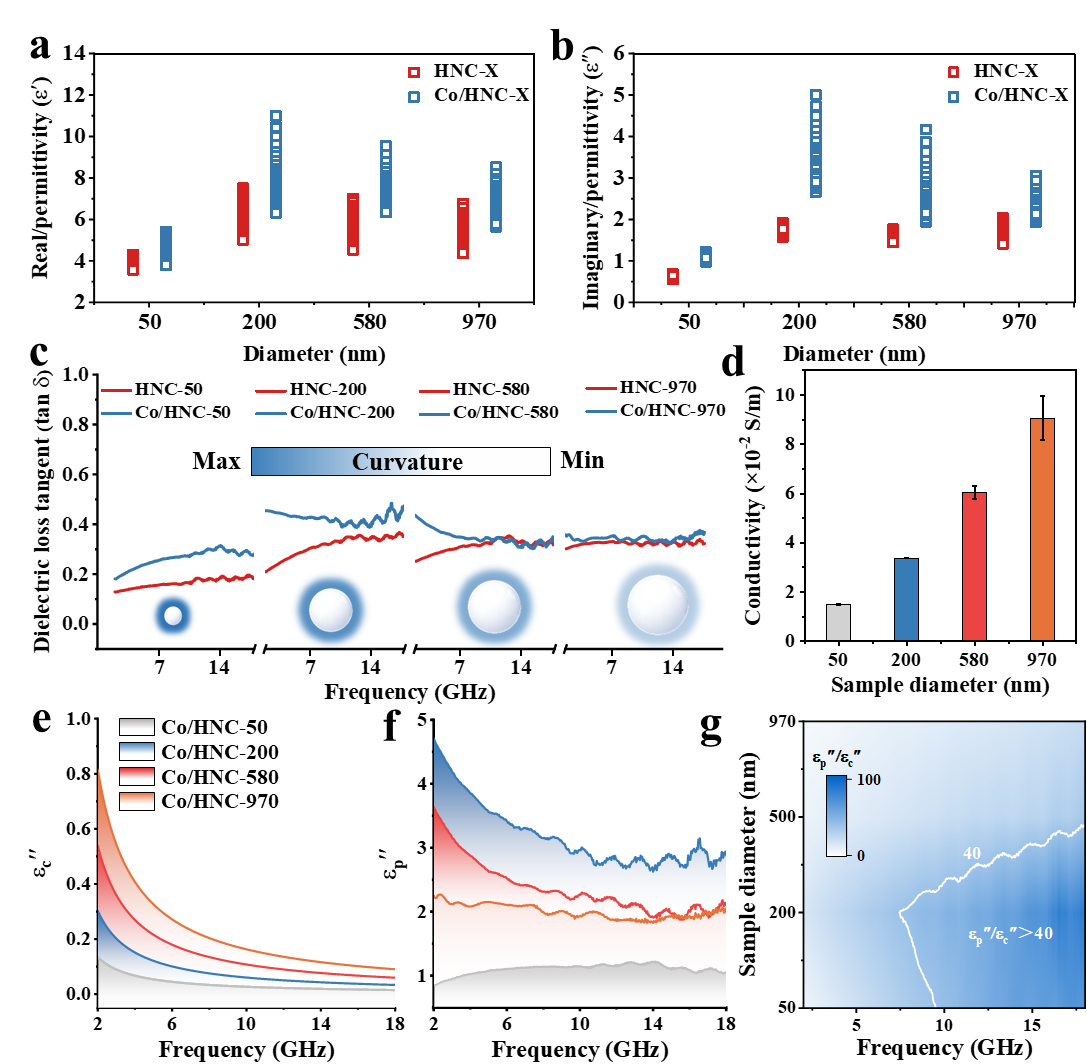


Figure S29. (a) Real and (b) imaginary part of complex permittivity. (c) Dielectric loss tangent, (d) electrical conductivity of samples dispersed in paraffin wax, (e) conduction loss, (f) polarization loss, and (g) polarization loss/conduction loss ratios of Co/HNC-X (X = 50, 200, 580, 970).


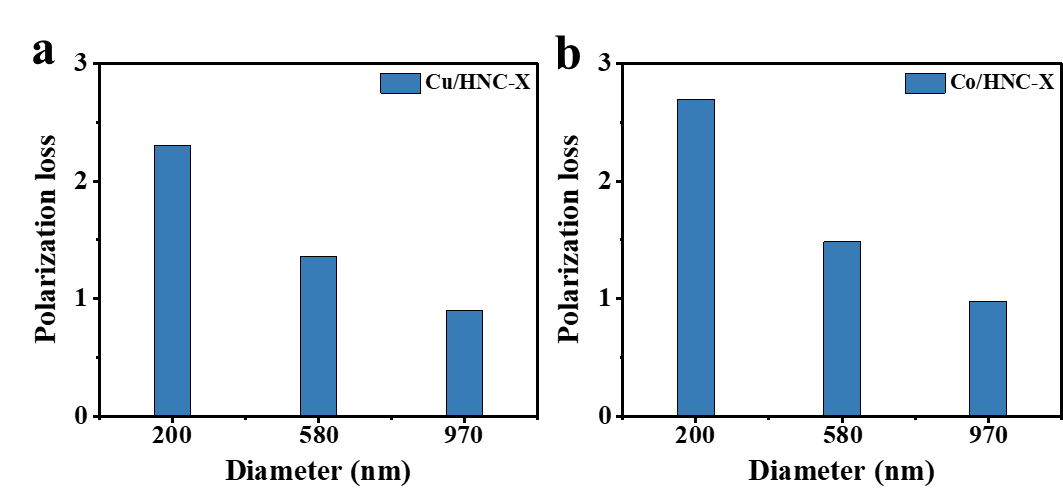


Figure S30. The calculated ε_c_″ and ε_p_″ (Data obtained by Python fitting).


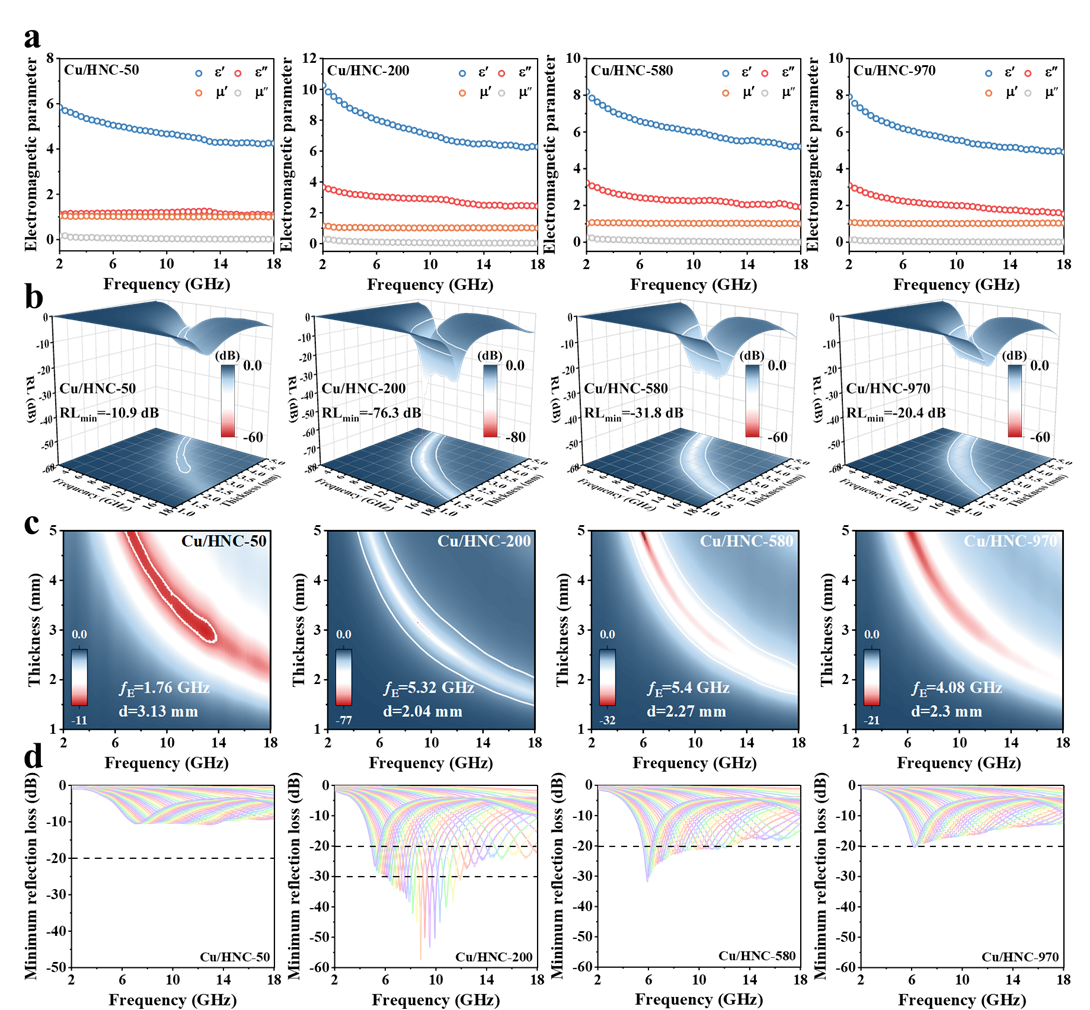


Figure S31. (a) Electromagnetic parameters, (b) 3D RL plots, (c) 2D RL plots and (d) RL curves (matching thickness: 1-5 mm; Increment: 0.1 mm) of Cu/HNC-X (X = 50, 200, 580, 970).


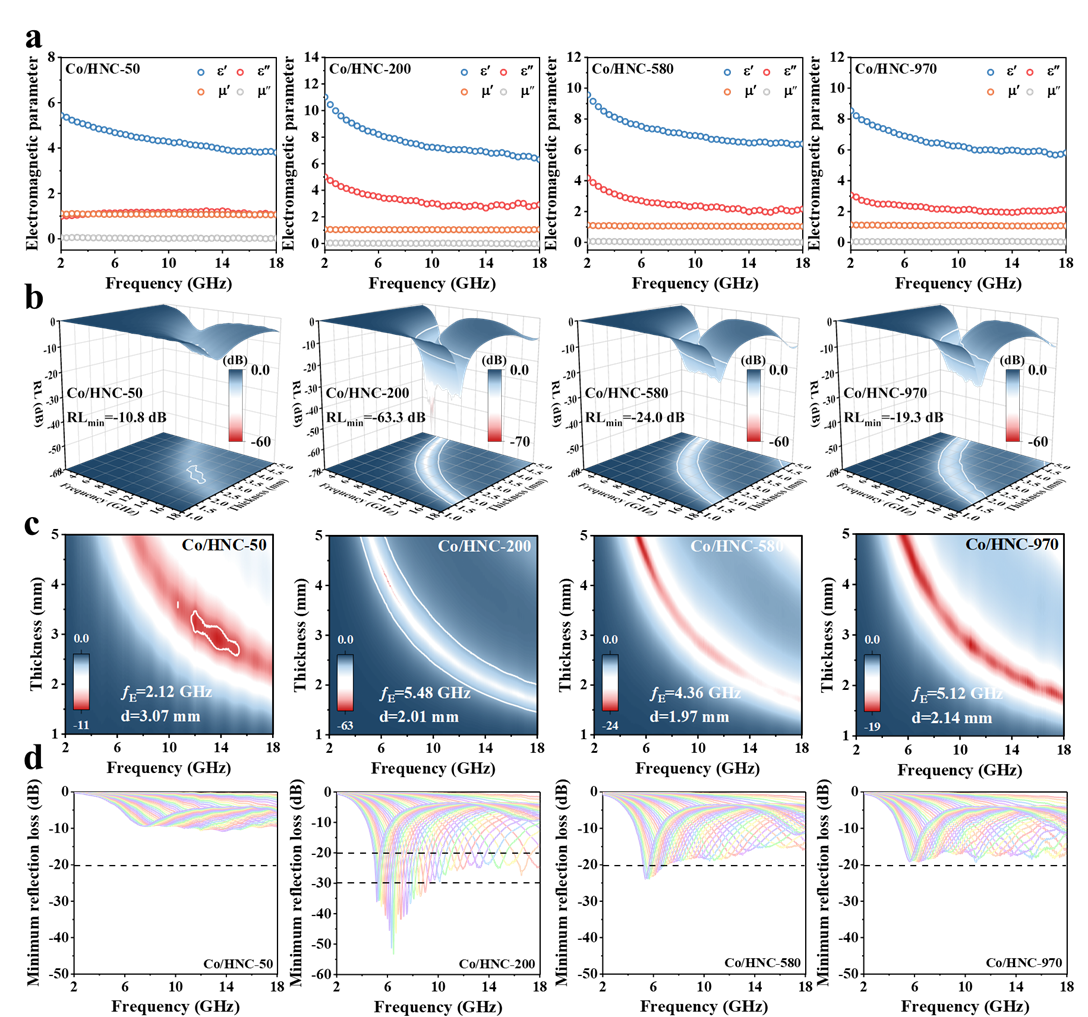


Figure S32. (a) Electromagnetic parameters, (b) 3D RL plots, (c) 2D RL plots and (d) RL curves (matching thickness: 1-5 mm; Increment: 0.1 mm) of Co/HNC-X (X = 50, 200, 580, 970).

**Note S6 RCS Simulation Results.**


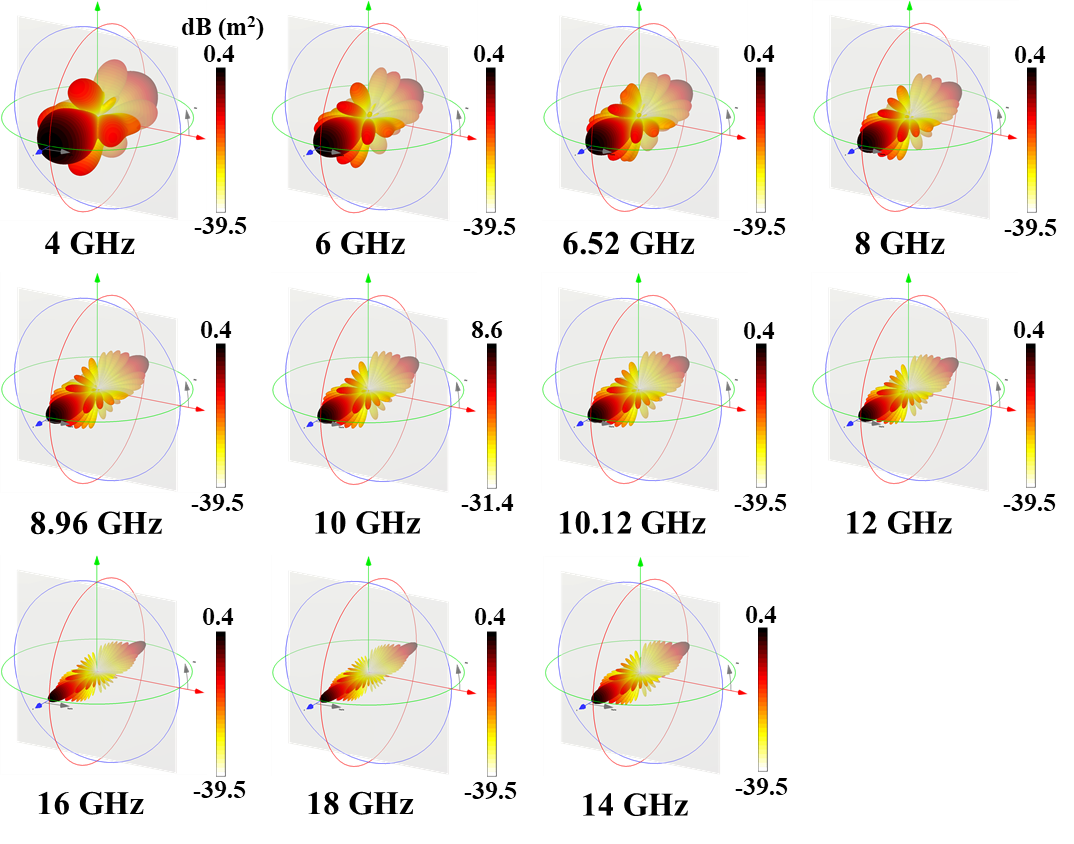


Figure S33. RCS simulation results of PEC at different frequencies.


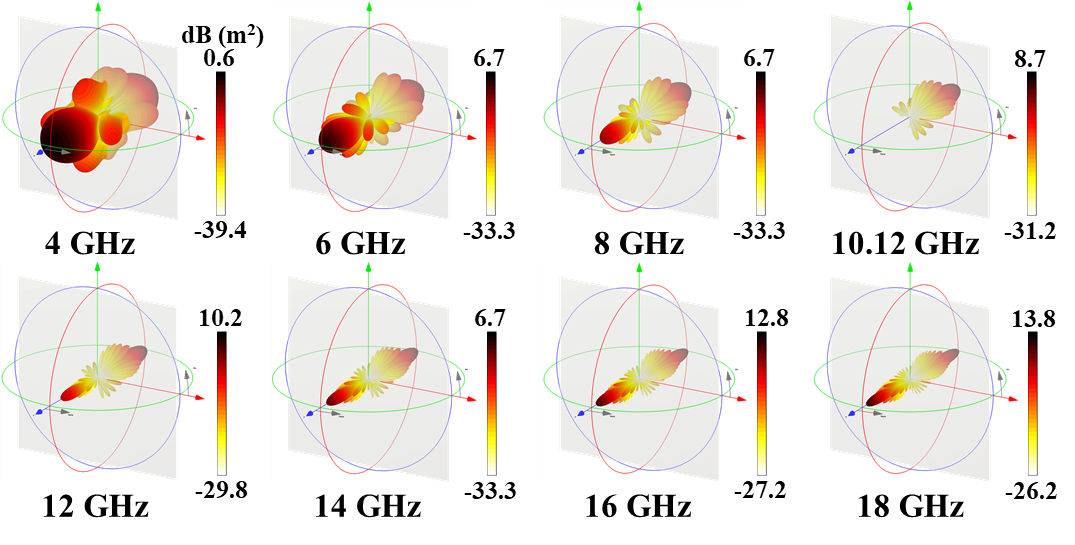


Figure S34. RCS simulation results of Ni/HNC-200 at different frequencies.


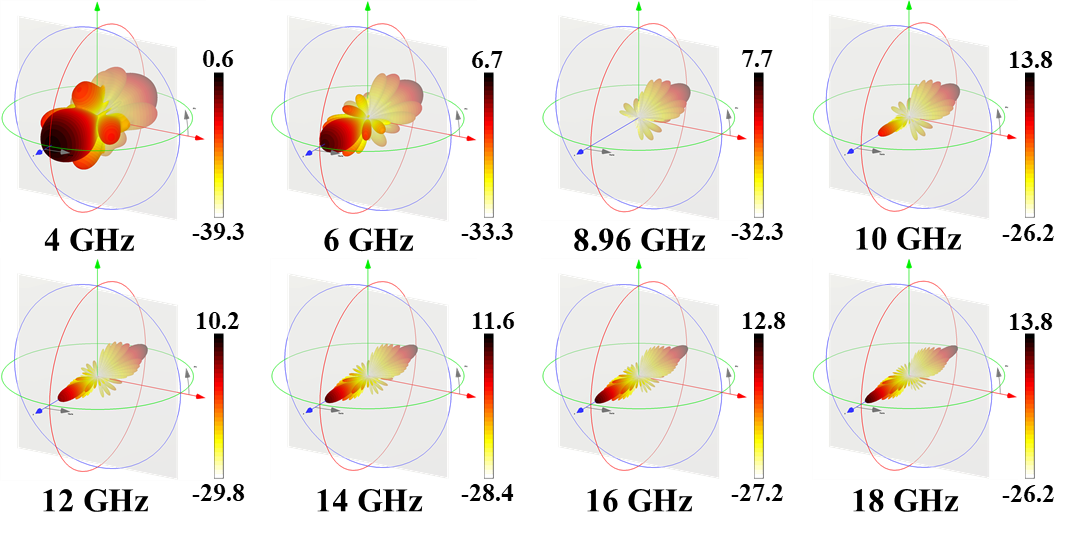


Figure S35. RCS simulation results of Cu/HNC-200 at different frequencies.


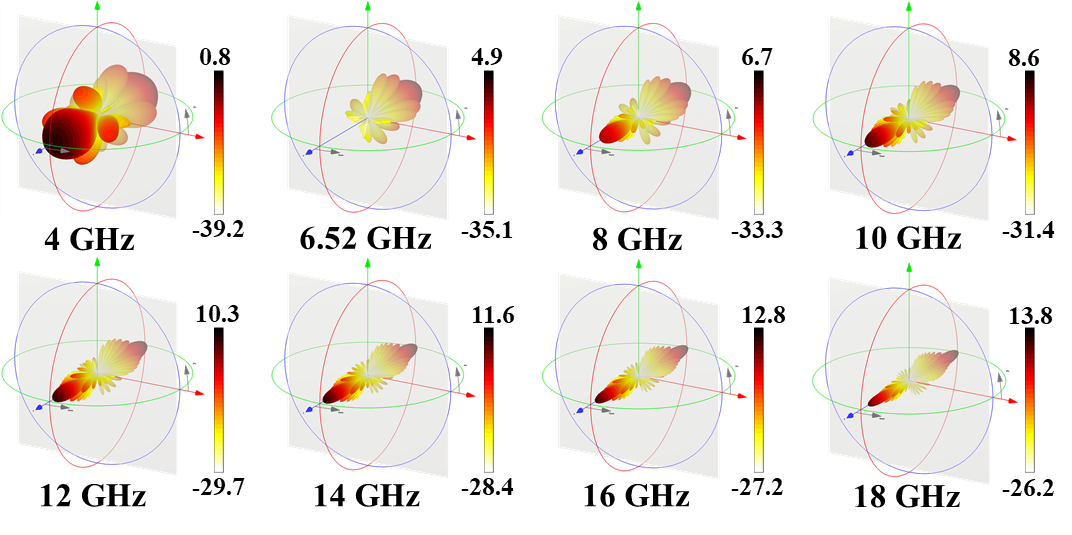


Figure S36. RCS simulation results of Co/HNC-200 at different frequencies.


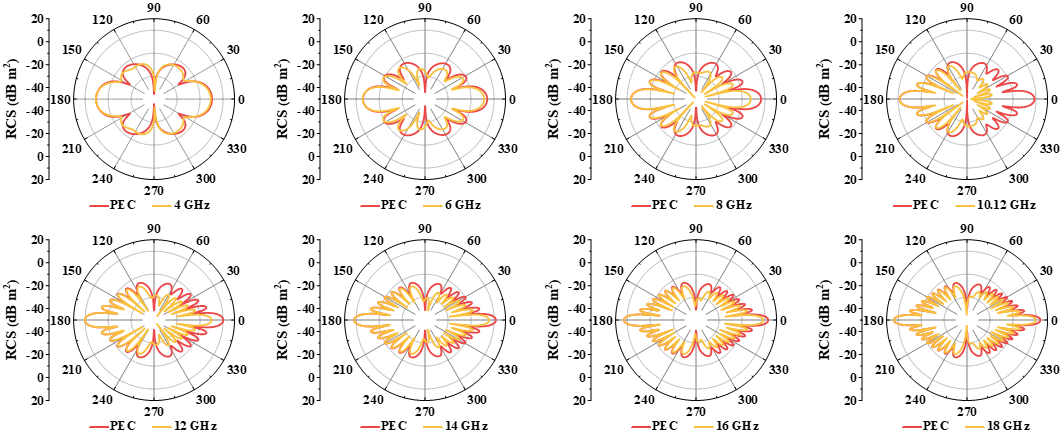


Figure S37. RCS radar maps of Ni/HNC-200 at different frequencies.


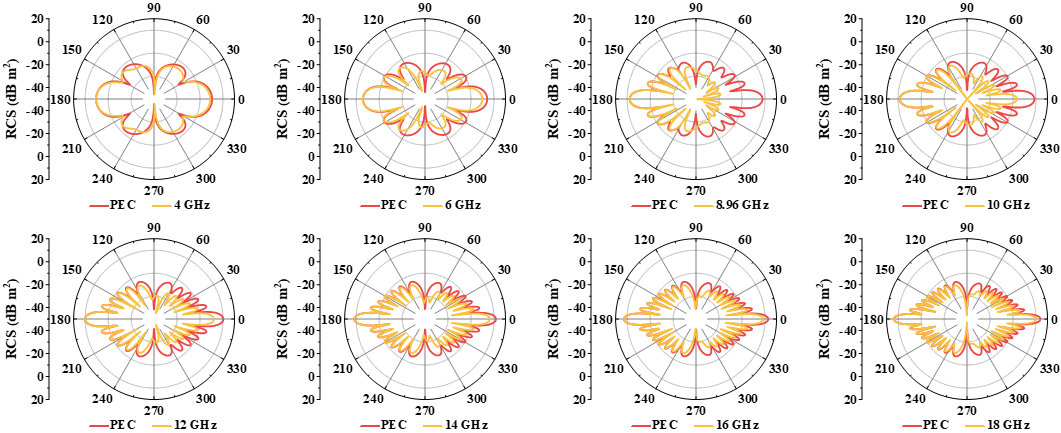


Figure S38. RCS radar maps of Cu/HNC-200 at different frequencies.


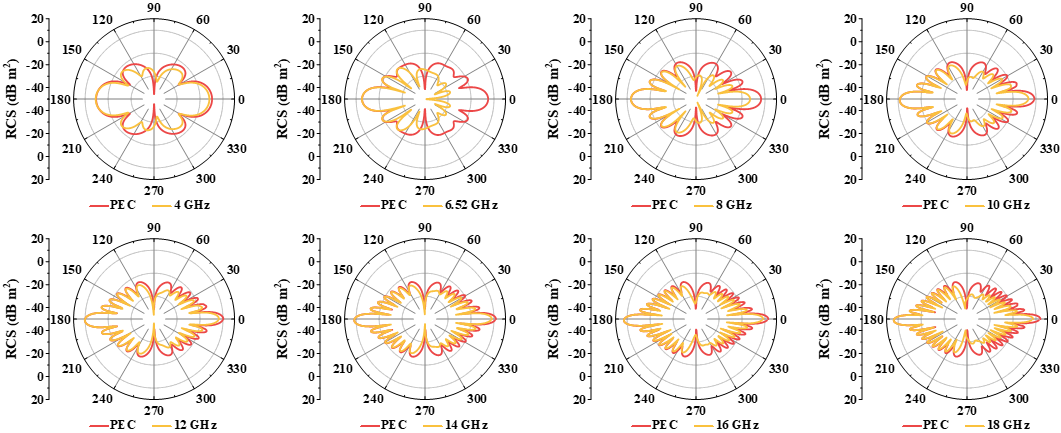


Figure S39. RCS radar maps of Co/HNC-200 at different frequencies.


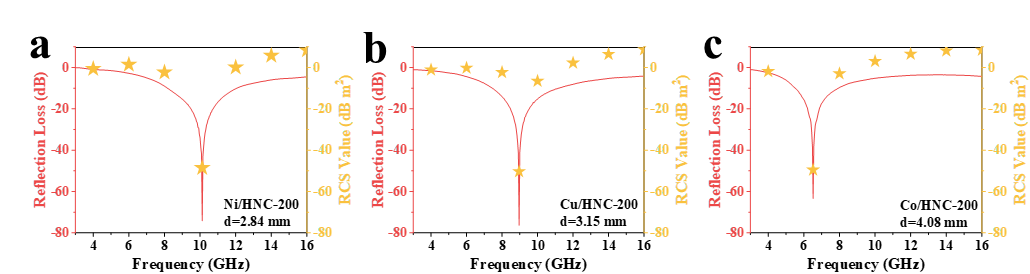


Figure S40. Corresponding relationship between RCS value and reflection loss of (a) Ni/HNC-200, (b) Cu/HNC-200, and (c) Co/HNC-200.

Figure S41. Simulated RCS curves of M/HNC-200 (M = Ni, Cu, Co).

**Note S7 Practical Applicability Assessment**


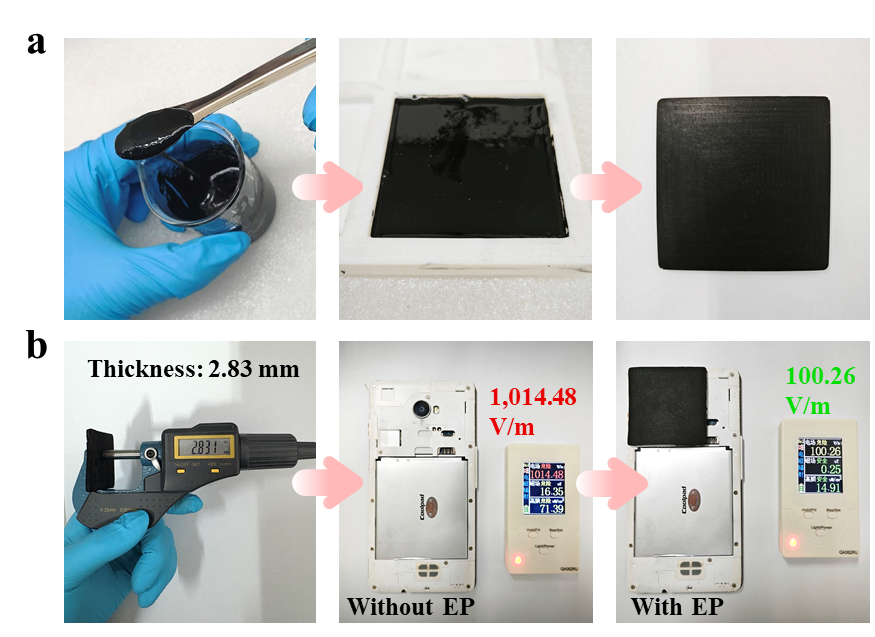


Figure S42. Electromagnetic patch testing. (a) Preparation and demonstration of wave absorbing patches. Ni/HNC-200 and polyvinylidene fluoride-hexafluoropropylene, were dissolved and dispersed in acetone to form a paste, which was then poured into a specific mold and air-dried. (b) Mobile phone covered with Ni/HNC-200 EP (Thickness: 2.83 mm) and schematic diagram of electromagnetic radiation detection with and without Ni/HNC-200 EP.

**Note S8 The Application Prospects of Curvature and Current Research Limitations**

The curvature effect has emerged as a cutting-edge and powerful strategy for tailoring material properties. Its application spans several advanced fields, including catalysis and energy conversion, where it plays a pivotal role. With its promise firmly recognized, curvature is now being intentionally incorporated into the architecture of electromagnetic wave (EMW) absorbing materials as a key design parameter. However, due to the unique nature of curvature as a physical parameter, the correlation mechanism between it and the dielectric properties of materials is difficult to quantitatively analyze, which severely restricts further development of related research. Consequently, the application of curvature manipulation in the design of wave-absorbing materials is still in its nascent stage and faces significant challenges. For example, strain-induced distortions in Fe–N bonds on highly curved Turing surfaces disrupt the symmetric charge distribution around Fe atoms, thereby enhancing dielectric loss. However, this work has significant limitations: (1) Uneven Turing surface leading to nonuniform curvature effects across the material surface; (2) The complexity of multi-component systems introduces multiple synergistic loss mechanisms (e.g., S-doping), complicating the isolation of nanocurvature's specific contributions; (3) A lack of effective experimental techniques and theoretical methods hinders the elucidation of how nanocurvature enhances EMW absorption. Therefore, it is imperative to develop innovative material design strategies, construct precise models to isolate individual loss mechanisms, and conduct in-depth analysis of how nanoscale curvature regulates the dielectric properties of materials by combining advanced characterization techniques with theoretical calculations.

Table S1. State-of-the art curvature engineering induced functions and applications

| **Representative studies on curvature in other fields** | | | | |
| --- | --- | --- | --- | --- |
|  | Material | Research contents | Research result | Ref |
| 1 | Fe/N-C | A new Fe/N–C catalyst with single Fe sites embedded within curved nanoprotrusions. The graphitized outer carbon layer of the nanoprotrusions can not only effectively weaken the binding strength of the oxygenated reaction intermediates, but also reduce the hydroxyl radical production rate | Fe/N-C achieves a record high power density of 0.75 W cm^−2^ under 1.0 bar H_2_–air with 86% activity retention after more than  300 hours of continuous operation | *Nature* **644,** 668-675 (2025) |
| 2 | HeterC-LCS | HeterC-LCS was synthesized by coupling the tip effect and nano-curvature effect to overcome the challenges of the scarcity of active sites and sluggish catalytic kinetics, which endowed it with higher local electric fields. Such synthesized catalysts behaved with an ultrahigh local electric field and H^+^ concentration in the catalytic interface | HeterC-LCS possessed an extremely low overpotential of 9.2 mV at 10 mA cm^−2^ and excellent mass and price activity of 72.8 A mg^−1^ and 6999.5 A dollar^−1^ at an overpotential of 100 mV | *Adv. Mater.* e15230 (2025) |
| 3 | SCSs | This work reports curvature-induced electron spin catalysis by using solid carbon spheres as catalysts, which were synthesized using positive curvature molecular hexabromocyclopentadiene as a precursor molecule, following a radical coupling mechanism. | The solid carbon spheres present excellent catalytic behaviour of oxidation coupling of amines to form corresponding imines with the conversion of >99 %, selectivity of 98.7 %, and yield of 97.7 %, which is attributed to the predominantly curvature-induced electron spin catalysis of carbon | *Angew. Chem. Int. Ed.* 64 e202412745 (2025) |
| 4 | Pd HC-Nds | This team has successfully synthesized a new class of Pd nanoneedles via a “close edges and open corners” process, with a magic angle of 60^o^ between the main trunk and branch. | A HCOOH productivity of ~250 mmol g^−1^ in 100 h while maintaining HCOOH selectivity over 99%. | *Nat. Comun.* **16** 6454 (2025) |
| 5 | Cu/Ni-MNC-T_60_ | In this work, a large number of high-curvature interfaces were identified in the as-synthesized mesoporous catalysts (Cu/Ni-MNC-T_x_, MNC = mesoporous nitrogen-doped carbon) through advanced three-dimensional reconstruction technologies. It was further confirmed that high-curvature interfaces can induce nondegenerate splitting of d-orbital energy levels in the affixing metal sites | Cu/Ni-MNC-T_60_ exhibited a significantly reduced apparent activation energy (from 102.7 to 52.8 kJ·mol^–1^) and remarkably promoted intrinsic activity (reduced energy barriers by 60%) in the hydrogenation of furfural | *J. Am. Chem. Soc.* 147 19166-19177 (2025) |
| 6 | Fe SA/NhcC | This team reports the superior catalytic activity for the oxygen reduction reaction (ORR) and enhanced performance for urea oxidation reaction (UOR) of single Fe atoms anchored on a highly curved N-doped carbon dodecahedron with concave morphology (Fe SA/NhcC) | The curvature of the carbon support helps to shorten the bond length of Fe−N, spatially redistributing the charges around the Fe and thereby lowering the d-band center toward optimal adsorption for oxygenated species | *Angew. Chem. Int. Ed.* 64 e202422920 (2025) |
| 7 | o-CNT-8 | This team reports the CNT curvature, or diameter, dependent ORR activity of o-CNT catalysts. | o-CNT-8 catalyst with the greatest surface curvature delivered the best 2e-ORR performance, even when compared to those of other recently reported carbon-based catalysts with similar oxygenous functional groups. They also observed a trend in catalyst stability performance that was dependent on the surface curvature | *ACS Catal.* 14 10928-10938 (2025) |
| 8 | HMHNs | A stable-monomicelle-assisted interface assembly method was developed for the precise synthesis of a regular 2D hierarchical mesoporous heterogeneous carbon/MXene nanosheet with a mesoscopic high surface pore curvature has been realized for the first time | The spherical/quasi-spherical interfaces can form transversely fully permeable channels, effectively preventing the loss of active sites when compared with the conventional flat 2D nanosheets with apparent stacking and aggregations. At the same time, they can also bring an obvious curvature effect to ensure the high-speed transport of ions. | *Matter* **8** 102164 (2025) |
| 9 | Fe-SAC-70 | They discover that uniformly tunable electric field modulation can be achieved using a model system of single-atom catalysts (SACs). These consist of M-N_4_ active sites hosted on a series of spherical carbon supports with varying degrees of nanocurvature. | Using in-situ Raman spectroscopy with a Stark shift reporter, we demonstrate that a larger nanocurvature induces a stronger electric field. Ni SACs with optimized nanocurvature achieved a high CO partial current density of ~400 mA cm^−2^ at >99% Faradaic efficiency for CO_2_ reduction in acidic media. | *Nat. Comun.* **15** 1719 (2024) |
| 10 | BM-PPy | This work introduces a novel parameter for designing capacitive deionization electrodes—the surface curvature parameter. And the surface curvature parameter was optimized to derive a surface curvature/electric field model, which was subsequently applied to the design of capacitive deionization electrodes. | Bicontinuous mesoporous polypyrrole with saddle-shaped high-curvature surfaces demonstrated an enhanced ion docking effect, which provided high salt adsorption capacity values of 262.7 mg g^−1^ at 1.2 V and 312.5 mg g^−1^ at 100 mA g^−1^, along with an ultra-long cycling life of over 2,000 cycles. | *Nat. Water* **2** 1195-1206 (2024) |
| 11 | RuNi/NC | This team propose an “on-site disruption and near-site compensation” strategy to reform the interfacial water hydrogen bonding network via deliberate cation penetration and catalyst support engineering. This concept is validated using tip-like bimetallic RuNi nanoalloys planted on super-hydrophilic and high-curvature carbon nanocages (RuNi/NC). | Tip-induced localized concentration of hydrated K^+^ facilitates optimization of interfacial water dynamics and intermediate adsorption. RuNi/NC exhibits low overpotential of 12 mV and high durability of 1600 h at 10 mA cm^‒2^ for alkaline HER | *Nat. Comun.* **15** 7179 (2024) |
| 12 | CoPc/SWCNTs | This study demonstrates that CNTs, as common supports for heterogeneous catalysts, not only serve as conductive substrates and prevent nanoparticle agglomeration but also regulate molecular catalyst performance by inducing stress through Å-scale distortions when molecular catalysts are introduced | CoPc/SWCNTs can achieve a *j*_MeOH_ of 66.8 mA cm^−2^ with an FE_MeOH_ of 31.3% in a CO_2_ atmosphere and a *j*_MeOH_ of 62.1 mA cm^−2^ with an FE_MeOH_ of 50.5% in a CO atmosphere | *Nat. catal.* **6** 818-828 (2023) |
| 13 | L-Cu_x_O-HC | This study presents a laser-based method to design bipyramid catalysts with tunable tip curvatures and abundant nanograins interfaces, resulting in improved kinetics and thermodynamics of multielectron reduction | The sharp tip geometry induces a localized strong electric field, improving electron transport and  ion concentration to regulate the reaction microenvironment in the  kinetic way. L-Cu_x_O-HC achieves a prominent C^2+^ partial current density of  665.9 mA cm−2 with an FE of 81% for CO_2_RR, and an NH_3_ yield rate of  81.83 mg h^−1^ mg^−1^ with a partial current density exceeding 600 mA cm^−2^ for NITRR. | *Nat. Comun.* **14** 7383 (2023) |
| 14 | Pt_1_/OLC | This team use onion-like nanospheres of carbon (OLC) to anchor stable atomically dispersed Pt to act as a catalyst (Pt_1_/OLC) for the HER. This highly curved structure causes electrons to accumulate around the Pt region, thereby inducing a local electric field and accelerating the catalytic kinetics | In acidic media, the performance of the Pt_1_/OLC catalyst (0.27 wt% Pt) in terms of a low overpotential (38 mV at 10 mA cm^−2^) and high turnover frequencies (40.78 H_2_ s^−1^ at 100 mV) is better than that of a graphene-supported single-atom catalyst with a similar Pt loading | *Nat. Energy* **4**, 512–518 (2019) |
| **Representative studies on curvature in EMW absorption fields** | | | | |
|  | Material | Research contents | Research result |  |
| 15 | Fe-SNC-T | An efficient strategy driven by the different thermal decomposition rates of two polymers isproposed to create Turing surface with highly curved stripes on hollow carbon  spheres (HCSs) for anchoring asymmetrically coordinated Fe-N_3_S_1_ moieties | Asymmetrically oordinated Fe-N_3_S_1_ moieties and the highly curved Turing surfaces efficiently broke the symmetric distribution of the charge near Fe atoms and significantly improved the dielectric loss property | *Adv. Funct. Mater.* **35**, 2413784 (2022) |

Table S2. The content of Ni in different samples measured by ICP-OES.

| **Sample** | **Ni (wt%)** |
| --- | --- |
| Ni/HNC-50 | 0.083 |
| Ni/HNC-200 | 0.075 |
| Ni/HNC-580 | 0.106 |
| Ni/HNC-970 | 0.104 |

Table S3. Fitting data of N 1s spectra (Percentage of different N types in samples of different diameters).

| Sample | Pyridinic N (%) | Pyrrolic N (%) | Graphtie N (%) | Metal-N (%) |
| --- | --- | --- | --- | --- |
| Ni/HNC-50 | 37.3 | 37.7 | 19.3 | 5.7 |
| Ni/HNC-200 | 38.2 | 37.8 | 18.7 | 5.3 |
| Ni/HNC-580 | 37.9 | 38.7 | 17.0 | 6.5 |
| Ni/HNC-970 | 37.2 | 38.6 | 17.0 | 7.2 |

Table S4. Parameters of the Ni K-edge EXAFS fitting results for samples.

| **Sample** | **shell** | **CN** | **R (Å)** | **ΔE_0_(eV)** | **σ^2^×10^3^(Å^2^)** | **R-factor** |
| --- | --- | --- | --- | --- | --- | --- |
| Ni/HNC-50 | Ni-N | 4.0 | 1.84 | -9.8 | 4.63 | 0.016 |
| Ni/HNC-200 | Ni-N | 3.9 | 1.84 | -8.0 | 3.14 | 0.020 |
| Ni/HNC-580 | Ni-N | 4.0 | 1.83 | -6.6 | 7.22 | 0.017 |
| Ni/HNC-970 | Ni-N | 3.8 | 1.86 | -7.8 | 5.56 | 0.014 |

Parameters of the Ni K-edge EXAFS fitting results for sample.

CN, coordination number; R, distance between absorber and backscatter atoms; ΔE_0_, the inner potential correction; σ^2^, Debye–Waller factor to describe the variance due to disorder (both lattice and thermal); R-factor is used to evaluate the quality of the fitting and the smaller value means more satisfied fitting. Error bounds that characterize the structural parameters obtained by EXAFS spectroscopy were estimated as N ± 20%; R ± 1%; σ^2^ ± 20%; ΔE_0_ ± 20%.

Table S5. Electromagnetic parameters and physical significance.

| **Electromagnetic parameter** | **Physical significance** |
| --- | --- |
| ε_r_ | Complex permittivity (the material's ability to respond to an electric field) |
| µ_r_ | Complex permeability (the materials’ response to a magnetic field) |
| ε′ and µ′ | Real part ( the ability to store energy) |
| ε″ and µ″ | imaginary part ( the ability to lose energy) |
| Z_in_ | impedance of absorbers |
| Z_0_ | impedance of free-space |
| f | incident EMW frequency |
| d | thickness of absorbers |
| c | velocity of light |
| ε_s_ | static permittivity |
| ε_∞_ | optical dielectric constant |
| ω | angular frequency |
| τ | polarization relaxation time |
| α | attenuation constant |

Table S6. Hirshfeld charge analysis of local Ni-N_4_ (charge quantity).

| **Sample** | **Ni** | **N4** | **Ni-N4** |
| --- | --- | --- | --- |
| **NiN_4_-P** | 0.0552 | 0.248 | 0.303 |
| **NiN_4_-15** | 0.0567 | 0.254 | 0.310 |
| **NiN_4_-10** | 0.0578 | 0.259 | 0.316 |
| **NiN_4_-5** | 0.0618 | 0.285 | 0.346 |

Table S7. Second-order hyperpolarizabilities (in au) of NiN_4_-P, NiN_4_-15, NiN_4_-10 and NiN_4_-5 in static electric field.

|  | **γ_xxxx_** | **γ_yyyy_** | **γ_zzzz_** | **γ_‖_** |
| --- | --- | --- | --- | --- |
| **NiN_4_-P** | 2716610 | 243068 | 675.1 | 753504.4 |
| **NiN_4_-15** | 2793410 | 251319 | 6017.0 | 778139.8 |
| **NiN_4_-10** | 1695830 | 229206 | 11843.5 | 593878.3 |
| **NiN_4_-5** | 14431200 | 1448580 | 98856.4 | 24706670 |

Table S8. Microwave absorption performance of other carbonaceous absorbers.

| **Types** | **Samples** | **Optimal RL**  **(dB)** | **d**  **(mm)** | **EAB**  **(GHz)** | **Ref.** |
| --- | --- | --- | --- | --- | --- |
| **Single-atom absorbers** | Fe-SNC-T | -40.62 | 2.0 | 4.88 | ^[[5]](#endnote-5)^ |
|  | Fe–SAs/NC | -52.5 | 1.7 | 4.8 | ^[[6]](#endnote-6)^ |
|  | Co/CNTs@ZSA/DCNs | -58.9 | 2.3 | 5.2 | ^[[7]](#endnote-7)^ |
|  | La-N_4_/ING | -48.5 | 1.8 | 4.1 | ^[[8]](#endnote-8)^ |
|  | Ni-SAs3/NC | -36.4 | 6.45 | 2.2 | ^[[9]](#endnote-9)^ |
|  | 3D Mn-NC | -46.2 | 2.0 | 4.7 | ^[[10]](#endnote-10)^ |
| **Hollow carbon-based absorbers** | HCNOs | -19.3 | 2.0 | 3.4 | ^[[11]](#endnote-11)^ |
|  | HCN-6 | -50.8 | 1.9 | 4.8 | ^[[12]](#endnote-12)^ |
|  | Carbon Capsule | -27.2 | 2.0 | 5.5 | ^[[13]](#endnote-13)^ |
|  | Hollow graphitic carbon nitride (HGCN) | -43.2 | 2.0 | 5.36 | ^[[14]](#endnote-14)^ |
|  | Graphene Nanocages (GN)_ | -51.1 | 1.45 | 4.4 | ^[[15]](#endnote-15)^ |
| **Other carbon-based absorbers** | rGO/SiC_nw_ | -19.6 | 3.0 | 4.2 | ^[[16]](#endnote-16)^ |
|  | Mn-Nx/NCNT | -31.48 | 1.7 | 4.15 | ^[[17]](#endnote-17)^ |
|  | Fe@NCNs-7 | -18.92 | 3.1 | 6.6 | ^[[18]](#endnote-18)^ |
|  | Fe-N/C-24 | -30.98 | 1.7 | 5.04 | ^[[19]](#endnote-19)^ |
|  | CoO/Co/N-CNTs | -52.3 | 2.2 | 5.28 | ^[[20]](#endnote-20)^ |
|  | CoMn@CN | -39.9 | 2.0 | 5.24 | ^[[21]](#endnote-21)^ |
|  | MnO@Co/C | -49.0 | 3.4 | 2.24 | ^[[22]](#endnote-22)^ |
| **This work** | Ni/HNC-200 | -74.1 | 1.97 | 5.24 | **This work** |
|  | Cu/HNC-200 | -76.3 | 2.04 | 5.32 |  |
|  | Co/HNC-200 | -63.3 | 2.01 | 5.48 |  |

Table S9. RCS values at θ =0° and average RCS values for different samples.

| **Sample** | **Frequency (GHz)** | **RCS_0°_** | **RCS_av_** | **RCS_min_** |
| --- | --- | --- | --- | --- |
| **Ni/HNC-200** | 10.12 | -48.55 | -24.2 | -70.49 |
| **Cu/HNC-200** | 8.96 | -50.33 | -24.0 | -73.15 |
| **Co/HNC-200** | 6.52 | -49.51 | -23.8 | -60.81 |

Movie S1.

Local Ni-N_4_ Dynamics of NiN_4_-5 under electric field.

Movie S2.

Local Ni-N_4_ Dynamics of NiN_4_-10 under electric field.

Movie S3.

Local Ni-N_4_ Dynamics of NiN_4_-15 under electric field.

Movie S4.

Local Ni-N_4_ Dynamics of NiN_4_-P under electric field.

**References**

1. . C. Liu, J. Lin, N. Wu, C. Weng, M. Han, W. Liu, J. Liu, Z. Zeng, *Carbon* **2025,** *223*, 119017. [↑](#endnote-ref-1)
2. . S. Zhang, D. Lan, J. Zheng, Z. Zhao, Z. Jia, G. Wu, *Cell Rep. Phys. Sci.* **2024**, *5*, 102206. [↑](#endnote-ref-2)
3. . M. Wei, K. Liu, Y. Wang, G. Zhang, Q. Liu, Q. Zhang, B. Zhang, *Small* **2024**, *20*, 2402632. [↑](#endnote-ref-3)
4. . M. Li, W. Zhu, X. Li, H. Xu, X. Fan, H. Wu, F. Ye, J. Xue, X. Li, L. Cheng, L. Zhang, *Adv. Sci.* **2022**, *9*, 2201118. [↑](#endnote-ref-4)
5. . Z. Ma, Y. Shen, X. Zhang, B. Li, Y. Chen, C. Zhu, *Adv. Funct. Mater.* **2025**, *35*, 2413784. [↑](#endnote-ref-5)
6. . Y. Su, B. Jiang, H. Shen, N. Yang, X. Tantai, X. Xiao, Y. Sun, L. Zhang, *Carbon* **2025**, *231***,** 119699. [↑](#endnote-ref-6)
7. . P. Liu, S. Zheng, Z. He, C. Qu, L. Zhang, B. Ouyang, F. Wu, J. Kong, *Small* **2024**, *20*, 2403903. [↑](#endnote-ref-7)
8. . Y. Shi, Z. Ma, X. Zhang, Z. Ma, F. Yan, C. Zhu, Y. Chen, *Adv. Funct. Mater.* **2024**, *34*, 2403508. [↑](#endnote-ref-8)
9. . H. Liang, G. Chen, D. Liu, Z. Li, S. Hui, J. Yun, L. Zhang, H. Wu, *Adv. Funct. Mater.* **2022**, *33*, 2212604. [↑](#endnote-ref-9)
10. . X. Zhang, Y. Shi, J. Xu, Q. Ouyang, X. Zhang, C. Zhu, X. Zhang, Y, Chen, *Nano-Micro Lett.* **2022**, *14*, 27. [↑](#endnote-ref-10)
11. . C. Ruan, Z. Li, D. Zhuang, X. Yuan, C. Liang, Y. Chang, H. Huang, L. Xu, M. Chen, *Carbon* **2020**, *161*, 622-628. [↑](#endnote-ref-11)
12. . C. Zhou, S. Geng, X. Xu, T. Wang, L. Zhang, X. Tian, F. Yang, H. Yang, Y. Li, *Carbon* **2016**, *108*, 234-241. [↑](#endnote-ref-12)
13. . J. Chen, P. Miao, E.E. Lin, T. Bai, S.K. Smoukov, J. Kong, *RSC Adv.* **2021**, *11*, 7954-7960. [↑](#endnote-ref-13)
14. . B. Fan, L. Xing, Q. He, F. Zhou, X. Yang, T. Wu, G. Tong, D. Wang, W. Wu, *Chem. Eng. J.* **2022**, *435*, 135086. [↑](#endnote-ref-14)
15. . C. Zhang, X. Li, Y. Shi, H. Wu, Y. Shen, C. Wang, W. Guo, K. Tian, H. Wang, *Adv. Opt. Mater.* **2022**, *10*, 2101904. [↑](#endnote-ref-15)
16. . M. Han, X. Yin, Z. Hou, C. Song, X. Li, L. Zhang, L. Cheng, *ACS Appl. Mater. Interfaces* **2017**, *9*, 11803–11810 (2017). [↑](#endnote-ref-16)
17. . Y. Wang, Y. Shi, X. Zhang, F. Yan, J. Zhang, X. Zhang, Y. Chen, C. Zhu, *Carbon* **2022**, *198*, 382-391. [↑](#endnote-ref-17)
18. . T. Gao, R. Zhao, Y. Li, Z. Zhu, C. Hu, L. Ji, J. Zhang, X. Zhang, *Adv. Funct. Mater.* **2022**, *32*, 2204370. [↑](#endnote-ref-18)
19. . J. Yu, J. Yu, T. Ying, X. Liu, X. Zhang, D. Han, *J. Alloys Compd.* **2020**, *838*, 155629. [↑](#endnote-ref-19)
20. . Z. Li, J. Liang, Z. Wei, X. Cao, J. Shan, C. Li, X. Chen, D. Zhou, R. Xing, C. Luo, J. Kong, *J. Mater. Sci. Technol.* **2024**, *168*, 114-123. [↑](#endnote-ref-20)
21. . Y. Wang, W. Zhong, S. Zhang, X, Zhang, C. Zhu, X. Zhang, X. Zhang, Y. Chen, *Carbon* **2022**, *188*, 254-2641. [↑](#endnote-ref-21)
22. . M. Liu, R. Tian, H. Chen, S. Li, F. Huang, K. Peng, H. Zhang, *J. Magn. Magn. Mater.* **2020**, *499*, 166289. [↑](#endnote-ref-22)
